# Effect of Genotype and Environment on *Salvia miltiorrhiza* Roots Using LC/MS-Based Metabolomics

**DOI:** 10.3390/molecules21040414

**Published:** 2016-03-26

**Authors:** Qi Zhao, Zhenqiao Song, Xinsheng Fang, Yuling Pan, Linlin Guo, Tian Liu, Jianhua Wang

**Affiliations:** State Key Laboratory of Crop Biology, Shandong Key Laboratory of Crop Biology, College of Agronomy, Shandong Agricultural University, Taian 271018, China; sdauzhaoq@163.com (Q.Z.); szqsdau@163.com (Z.S.); xinshf@126.com (X.F.); mingwangxingmomo@163.com (Y.P.); guolianlin2013@163.com (L.G.); tea1022@163.com (T.L.)

**Keywords:** *Salvia miltiorrhiza* Bunge, secondary metabolites, metabolite profiling, LC-MS, multivariate analysis

## Abstract

*Salvia miltiorrhiza* (*S. miltiorrhiza*) Bunge is broadly used as herbal medicine for the clinical treatments of cardiovascular and cerebrovascular diseases. Despite its commercial and medicinal values, few systematic studies on the metabolome of *S. miltiorrhiza* roots have been carried out so far. We systematically described the metabolic profiles of *S. miltiorrhiza* using high pressure liquid chromatography mass spectrometry (LC/MS) in conjunction with multivariate statistical analyses, aimed at monitoring their biological variations of secondary metabolites related to three locations and four *S. miltiorrhiza* genotypes. A total of 40 bioactive constituents were putatively annotated in *S. miltiorrhiza* root samples. This study found that both the same *S. miltiorrhiza* genotype growing at three different locations and four *S. miltiorrhiza* genotypes growing at the same location had significant metabonomic differences identified by the principal component analysis (PCA) approach. By using orthogonal projection to latent structure with discriminant analysis (OPLS-DA), 16 and 14 secondary metabolites can be used as potential location-specific and genotype-specific markers in *S. miltiorrhiza*, respectively. The specificity of LC/MS profiles offered a powerful tool to discriminate *S. miltiorrhiza* samples according to genotypes or locations.

## 1. Introduction

*Salvia miltiorrhiza* (*S. miltiorrhiza*) Bunge is broadly used as Chinese medicine for the clinical treatments of cardiovascular and cerebrovascular diseases, chronic renal failure, neurasthenic insomnia, dysmenorrheal, and liver cirrhosis [1]. One of the traditional Chinese medicine preparations (TCMPs), Compound Danshen dripping pills, has passed US Food and Drug Administration phase II clinical trials because of its efficacy in curing cardiovascular disease. *S. miltiorrhiza* is a widely cultivated crop in many provinces in China with enormous industrial consumption. Approximately 80 million kilograms of *S. miltiorrhiza* are required for food and pharmaceutical industry each year [2].

Phenolic acids and tanshinones are two major bioactive compounds in *S. miltiorrhiza*. Phenolic acids are responsible for many pharmaceutical effects (e.g., antioxidation, and the scavenging of free radicals) [3]. Tanshinones also exhibit a variety of therapeutic actions (e.g., antibacterial and antitumor activities) [4]. These have been ascribed to their inhibition of the Aurora A kinase and the hypoxia-inducible factor-1 [5,6]. During production, *S. miltiorrhiza* species show variations in the contents of bioactive compounds according to geographical origins [7,8], metabolic engineering [9], cultivation ages [10], crop management practices, and various environmental conditions [11,12,13]. The quality variations inevitable affect its pharmacological activities and tonic functions [14], so production protocols for the cultivation of stable-yield and stable-quality *S. miltiorrhiza* are needed.

Metabolomics has been widely used to detect subtle and potential changes in all the metabolites of plants and animals, which links phenotypes to their corresponding genotypes. Plant metabonomic analysis has been established as an important molecular phenotyping approach for comprehending gene functions [15], plant variations [16], environment sciences [17], and agricultural production systems in agricultural and industrial biotechnology. Applications of the metabolomics have also been displayed in quality controls of medicinal plants, e.g., *Panax ginseng*, and *Artemisia annua* L. [15,18].

With specific regard to different locations, different growing conditions in *Marrubium vulgare* L. (Lamiaceae) might induce differences in phenolic acids content and antioxidant activity [19]. Similarly, different geographical origins in *S. miltiorrhiza* samples might induce differences in primary and secondary metabolites [7,8].

With regard to different genetic background, different cultivars in *Ocimum* species (Lamiaceae) might induce differences in flavones content [20]. In two *S. miltiorrhiza* genotypes, the differences in rosmarinic acid accumulation were mainly due to genetic influences [21].

With regard to the genotype-by-environment interaction, terpenoids showed significant differences between two *Curcuma* species, whereas geographical location had little effect [17]. Furthermore, phenolic compounds of olive oils differed significantly among locations and cultivars [22].

Previous studies have demonstrated some quality variations for *S. miltiorrhiza* genotypes and the same genotype grown at different environment [9,12]. However, those studies only consider several selected secondary metabolites, and they do not evaluate the genotype-by-environment interaction and holistic metabonomics in *S. miltiorrhiza*. In the present study, the effects of genetic background and environment were simultaneously investigated using the LC-MS method in conjunction with multivariate statistical analyses. The objective of this study is to find out the statistically significant metabolites associated with different genotypes and locations, respectively. These metabolites can be used as potential genotype- and location-specific markers in *S. miltiorrhiza*. The results of this study may help to better understand the quality variation due to environment and genotype in *S. miltiorrhiza*.

## 2. Results and Discussion

### 2.1. Identification of Metabolites

In the present study, the constituents of *S. miltiorrhiza* were tentatively identified by comparison of their exact molecular weight, MS^2^ fragmentation and UV spectra with published papers described by Fang *et al.*, Dai *et al.*, and Liu *et al.* [23,24,25,26]. Moreover, eight metabolites were accurately identified by comparing their spectral absorption and accurate retention time with reference compounds under the same experimental condition [8].

Finally, in total, 40 out of the 66 metabolites, including 16 phenolic acids, 20 tanshinones and four other compounds (*i.e*., luteolin, royleanone-4, 7α-hydroxyallyl-royleanone, and β-sitosterol), could be putatively identified in *S. miltiorrhiza* (Table 1). Most of the *m/z* data were [M − H]^−^, [M + H]^+^, and [M + Na]^+^. Phenolic acids had good responses in both negative and positive ion modes, while tanshinones, royleanone-4,7α-hydroxyallyl-royleanone, and β-sitosterol only had good responses in positive ion mode, which was consistent with the result obtained from Dai *et al.* (Table 1) [25].

### 2.2. Effect of Environment, Multivariate Analysis

**Figure 1:**
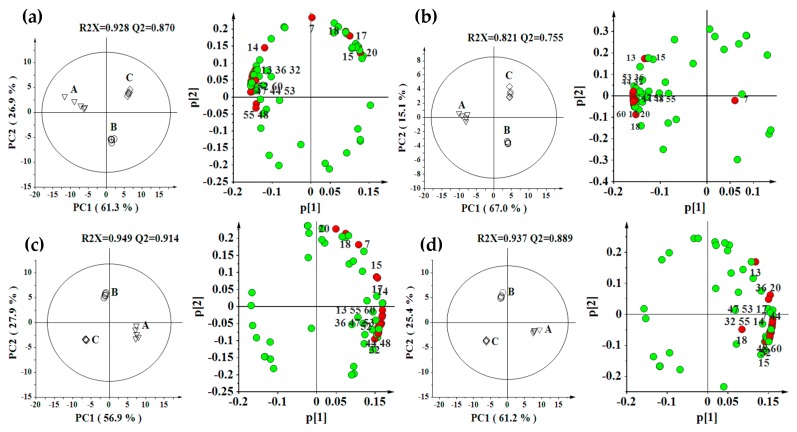
PCA scores plots and loading plot derived from LC-MS data for *S. miltiorrhiza* extracts from three different locations (A, Zhuyang; B, Changqing; C, Taian) for each genotype ((**a**) Genotype 1; (**b**) Genotype 2; (**c**) Genotype 3; and (**d**) Genotype 4). Sixteen red colored metabolites in loading plot were the location-specific metabolites that we obtained from OPLS-DA: 7, Salvianolic acid F; 13, Salvianolic acid I; 14, Salvianolic acid E; 15, Rosmarinic acid; 17, Lithospermic acid; 18, Prolithospermic acid derivative; 20, Salvianolic acid B; 32, Tanshinone IIB; 36, Trijuganone C; 44, 15,16′-dihydrotanshinone I; 47, Methyltanshinonate; 48, Trijuganone B; 52, Cryptotanshinone; 53, Tanshinone I; 55, 1,2′-dihydrotanshinone I; and 60, Tanshinone IIA.

In order to determine the effect of growing environment, we used the unsupervised principal components analysis (PCA) to observe the variation in different *S. miltiorrhiza* locations for each genotype (Figure 1). Samples from Zhuyang location were separated completely from samples at Changqing and Taian locations along PC1 which accounted for 56.9%–67.0% of the total variance for each genotype, and samples from Changqing and Taian locations were separated along PC2 which accounted for 15.1%–27.9% of the total variance, indicating that there was a remarkable metabonomic difference between Zhuyang and two other locations (Figure 1). Tight intragroup clustering indicated that technical error was lower than environmental influence.

**Figure 2:**
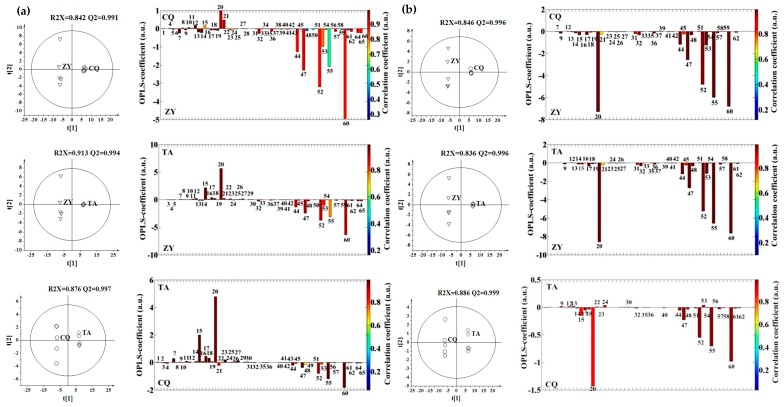

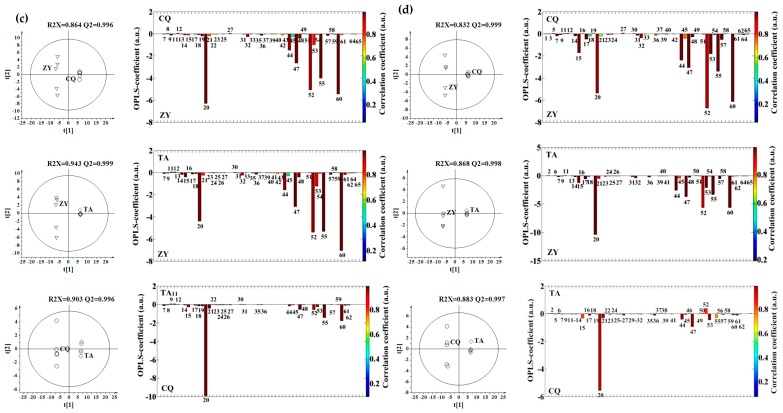
OPLS-DA scores plots (**left**) and coefficient-coded loadings plots (**right**) deriver from LC-MS data for *S. miltiorrhiza* extracts obtained from three different locations (ZY, Zhuyang; CQ, Changqing; TA, Taian) for each genotype ((**a**) Genotype 1; (**b**) Genotype 2; (**c**) Genotype 3; and (**d**) Genotype 4): 7, Salvianolic acid F; 13, Salvianolic acid I; 14, Salvianolic acid E; 15, Rosmarinic acid; 17, Lithospermic acid; 18, Prolithospermic acid derivative; 20, Salvianolic acid B; 32, Tanshinone IIB; 36, Trijuganone C; 44, 15,16′-dihydrotanshinone I; 47, Methyltanshinonate; 48, Trijuganone B; 52, Cryptotanshinone; 53, Tanshinone I; 55, 1,2′-dihydrotanshinone I; and 60, Tanshinone IIA.

The orthogonal projection to latent structure with discriminant analysis (OPLS-DA) was used to highlight the group separation (Figure 2), and demonstrated an enormous separation of different locations along the t [1] axis. Good model quality was showed by the values of R^2^X and Q^2^.

**Figure 3:**
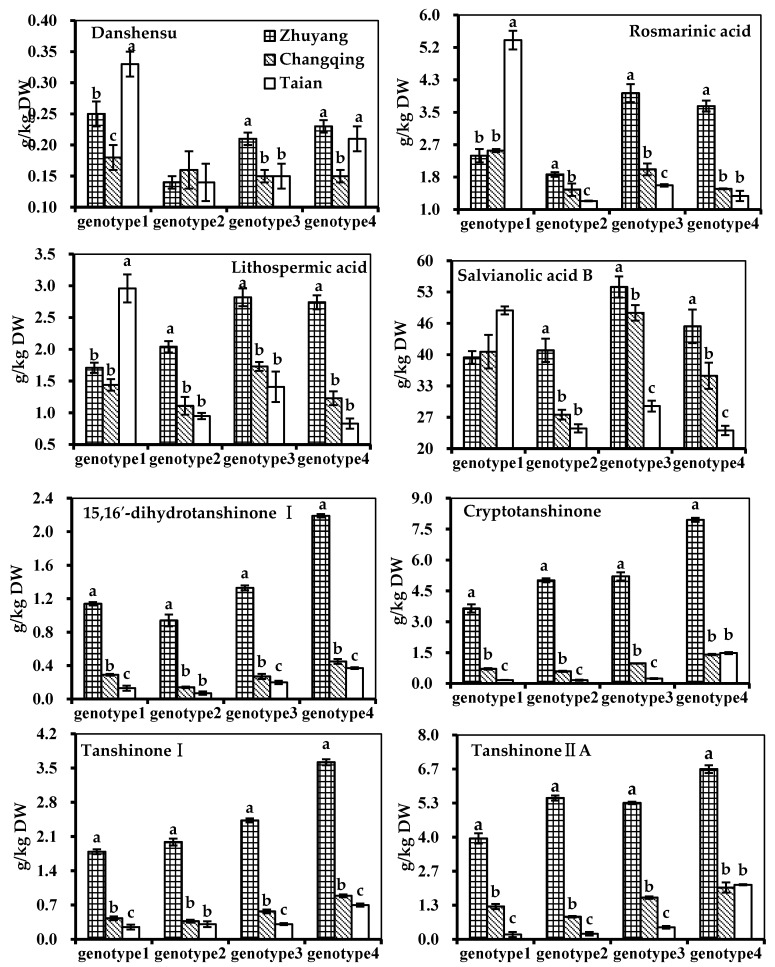
Absolute quantification of eight selected metabolites. Concentrations are given in g/kg DW. Data are shown as mean of concentration ± SD. Values between three locations having different lowercase letters (a, b, and c) are significantly different at *p* < 0.001/3 (Bonferroni correction).

Figure 2a shows OPLS-DA scores and loading plots between locations in Genotype 1 samples. Compared with Changqing samples, the Zhuyang samples contained more phenolic acids (e.g., 7, 13, 14, 17, and 18), and tanshinones (e.g., 32, 36, and 47) but less other phenolic acids (e.g., 4, 10, and 20) in Genotype 1 (Figure 2a). Nevertheless, this was not completely agreeable to the absolute quantification (Figure 3) because of different UV response coefficient for different metabolites [24]. This suggested that absolute quantification was probably necessary when LC/MS were used for metabolite profiling. In Genotype 1, Zhuyang samples contained more tanshinones (e.g., 32, 44, 47, 48, 52, and 60) but less other phenolic acids (e.g., 7, 15, 17, 18, and 20) than Taian samples (Figure 2a). This result was in agreement with the accurate quantification in Genotype 1 (Figure 3). Furthermore, Changqing samples included more tanshinones (e.g., 44, 47, 52, 55, and 60) than Taian samples together with less phenolic acids (e.g., 7, 14, 15, 17, 18, and 20) in Genotype 1 (Figure 2a). This was broadly consistent with the quantification (Figure 3). Thus, in Genotype 1, Zhuyang location showed higher levels in tanshinones, and Taian location showed higher levels in phenolic acids (Figure 2a).

Interestingly, unlike Genotype 1, Genotypes 2, 3 and 4 exhibited the same change regularity for each paired locations (Figure 2), so the metabolites with significant difference in Genotypes 2, 3 and 4 were simultaneously analyzed for each paired locations. Detailed loading analyses for Genotype 2, 3 and 4 revealed that compared with Changqing and Taian location, Zhuyang samples contained more phenolic acids (e.g., 14, 15, 17, and 20) and tanshinones (e.g., 32, 44, 47, 48, 52, 55, and 60) (Figure 2b,c,d) which was consistent with the quantification (Figure 3). Changqing samples also had more phenolic acids (e.g., 14, 17, and 20) and tanshinones (e.g., 44, 47, 55 and 60) than Taian samples in Genotypes 2, 3 and 4 (Figure 2b,c,d) which was not completely agreeable with the quantification (Figure 3) due to the different UV response coefficient. Thus, in Genotypes 2, 3 and 4, Zhuyang location showed higher levels in both phenolic acids and tanshinones than Changqing and Taian locations (Figure 2). Finally, tanshinones were negatively correlated with phenolic acids in Genotype 1 samples, and positively correlated with phenolic acids in Genotype 2, 3 and 4 samples.

In previous studies, salvianolic acid B, rosmarinic acid, cryptotanshinone, tanshinone I and tanshinone IIA were optimized as makers of different locations in 74 *S. miltiorrhiza* samples [8]. Malonate and succinate were used as the key markers for distinguishing the geographical origin of *S. miltiorrhiza* based on the regulation of the root respiratory rates [7].

Through our research, the correlation coefficients of metabolites having significant differences (*p* < 0.001) were showed in Table S1. The top 16 major contributors including salvianolic acid F, salvianolic acid I, salvianolic acid E, salvianolic acid B, rosmarinic acid, lithospermic acid, prolithospermic acid derivative, tanshinone IIB, tanshinone I, tanshinone IIA, trijuganone C, 15,16′-dihydrotanshinone I, methyltanshinonate, trijuganone B, cryptotanshinone, and 1,2′-dihydrotanshinone I, were found to have significant intergroup differences for different locations (Tables S1 and S2). These metabolites could be used as potential location-specific markers in *S. miltiorrhiza*. Great differences of these location-specific metabolites were also observed by PCA loading plots (Figure 1).

In case of phenolic acids, targeted analysis was used to explore the influence of environment (Figure 3). Inconsistent change regularity for the four genotypes to different locations was found in our studies. Except Genotype 1, rosmarinic acid, lithospermic acid and salvianolic acid B contents followed the order at different locations: Zhuyang > Changqing > Taian (Figure 3). Thus, the phenolic acid content was significantly affected by growing locations. Genotype 1 was different from the other three genotypes, and it could remain the salvianolic acid B relative stability (39.23 g/kg–49.13 g/kg) (Figure 3).

In case of tanshinones, targeted analysis was used to explore the influence of environment (Figure 3). The contents of four tanshinones (15,16′-dihydrotanshinone I, cryptotanshinone, tanshinone I, and tanshinone IIA) were highly influenced by locations (Figure 3). *S. miltiorrhiza* grown in Zhuyang had clearly higher tanshinones content than their counterparts grown in Changqing and Taian locations (Figure 3).

Many studies showed that secondary metabolites of plants were significantly influenced by geographical factors and environmental conditions. In *S. miltiorrhiza*, phenolics and tanshinones accumulation can be stimulated by both biotic and abiotic elicitors. The contents of phenolic acids and tanshinones increased under water-stress, nitrogen (N)-stress, and high Cd^2+^ conditions in *S. miltiorrhiza* [2,11,13].

This study might be explained by the plant–soil interactions (e.g., plant–N interactions and plant–water interactions) in different locations. In plant–N interactions, Zhuyang location had less soil fertility (sandy loam, 5.1 g/kg soil organic carbon, 34.88 mg/kg available N) and much bioactive components, while Taian location had much soil fertility (loam, 15.4 g/kg soil organic carbon, 55.28 mg/kg available N) and less bioactive components (Figure 3). These negative effects of N application on the contents of bioactive components were in agreement with those reported in *Ocimum basilicum* L. and *Chrysanthemum boreale* M. [27,28]. Previous study reported that plant growth, danshensu, salvianolic acid B, cryptotanshinone, tanshinone IIA responded negatively to increasing N availability, suggesting that *S. miltiorrhiza* was not a nitrophile [2]. Our results indicated that plant growth was promoted while secondary metabolites were inhibited with the increase of nitrogen, which was different from previous study [2]. This difference might come from environmental variability, such as nitrogen, temperature and total rainfall. Our study might be unique to *S. miltiorrhiza* grown in Shandong province, China. For example, this difference might come from the content of organic carbon in the soil. In our study, the scope of organic carbon was 5.1–15.4 g/kg, while the soil in previous study contained more organic carbon (>13.3 g/kg) because of fertilization [2]. Combining with previous study, plant growth might increase at first (our study) and then decreased (previous study) with the increase of organic carbon.

With respect to plant–water interactions, Zhuyang location had less water (sandy loam) and much bioactive components, while Taian location had much water (loam) and less bioactive components (Figure 3). Previous research showed that drying-induced stress involved both its primary and secondary metabolites (e.g., proline, sucrose, and salvianolic acid B) in *S. miltiorrhiza* [25]. Proline was regarded as a scavenger for reactive oxygen species (ROS), and sucrose was the key ingredient of photosynthetic and nonphotosynthetic carbon translocation [25]. In our studies, the metabolic pathways of phenolic acid and tanshinone were activated simultaneously as defensive response of *S. miltiorrhiza* to N-stress and water-stress in Genotypes 2, 3, and 4, so tanshinones were positively correlated with phenolic acids in Genotypes 2, 3 and 4 samples.

There were several possible explanations for this observation. First, the carbon-nutrient balance and growth-differentiation balance hypotheses predicted a balance between growth and defence. Because environmental stress reduced plant growth (Figure S1a), the carbon fixed during assimilation can be applied to the accumulation of secondary metabolites. Previous studies found that genes related to stress response, and terpenoid metabolism were greatly up-regulated, and genes related to growth and development were mostly down-regulated by yeast extract and Ag+ treatments in *S. miltiorrhiza* [29]. Second, environmental stress could alter enzyme activity in *S. miltiorrhiza*. Water stress and N stress might induce oxidative stress because of the formation of ROS [11]. To protect plants against the injury of ROS, *S. miltiorrhiza* accumulated secondary metabolites by altering their enzyme activities (Figure S1b) [25]. Figure S1b showed that the higher phenolic acids accumulation might be a result of the up-regulation of several co-expressed genes in this metabolic pathway. Further gene expression analysis were warranted to consider in future studies. Several investigations had shown that phenolic acids and tanshinones in *S. miltiorrhiza* had antioxidant activities. Thirdly, some important signal molecules (e.g., methyl jasmonat, and nitric oxide), which could increase tanshinone production [30], might also increase due to environmental stress in our studies. Previous research found that the jasmonic acid carboxyl methyltransferase, which directly produces methyl jasmonat, was strongly increased after yeast extract and Ag+ treatments [29].

Among the phenolic acids, salvianolic acid B was the main water-soluble component. Genotype 1 could remain the salvianolic acid B relative stability (39.23–49.13 g/kg) compared to three other genotypes, indicating that Genotype 1 was possibly related to resistances (Figure 3). Some resistant genes, which were involved in the water stress, had been found in *S. miltiorrhiza* [31]. Furthermore, in the statistics of morphological characters and disease resistance (Table S3), we also found that Genotype 1 might have stronger resistance because of its thick stalk, developed root system and resistance to disease. Previous study reported that phenolic acids had greater resistance compared with tanshinones [12]. Thus, in Genotype 1, tanshinones could be stimulated by environmental stress, while phenolic acid could not be stimulated by the same environment because of its resistance. The different responses of phenolic acids contents in different genotypes suggested the functional complexity of secondary metabolism in self-protection.

In previous studies, people had observed the inconsistency of the change regularity between phenolic acids and tanshinones under water-stress, and Ag^+^ conditions in *S. miltiorrhiza* [11,12]. Ag^+^ significantly increased the contents of cryptotanshinone, dihydrotanshinone I, tanshinone I, and tanshinone IIA, and decreased that of danshensu, and salvianolic acid B [12]. Furthermore, concerning the phenolic acids contents of the olive oils between different locations, significant differences were highlighted in Peranzane cultivar, whereas no difference was observed in Ogliarola cultivar [22]. Although phenolic acids could not be simulated by the environmental stress in Zhuyang location in Genotype 1, plant growth was inhibited (Figure S1). Therefore, the contents of danshensu, rosmarinic acid, and lithospermic acid might decrease at stress conditions in Genotype 1 because of the reduction of the assimilation.

In general, production protocols for the cultivation of high-yielding and high-quality *S. miltiorrhiza* were needed, but sometimes we could not satisfy all the demands. Although Zhuyang location had the lowest yield, it had the highest secondary metabolites content in Genotype 2, 3 and 4. Although Taian location did result in high yields, the quality might be not high enough to conform to the standards required by the Chinese Pharmacopoeia (The Pharmacopoeia Commission of PRC, 2010) to achieve satisfactory therapeutic effects. Previous study showed that variation in *S. miltiorrhiza* concentrations influence the therapy of the neural differentiation efficiency [1]. However, this finding of the resistance in Genotype 1 should be the key to resolve conflict between high-yielding and high-quality in commercial cultivation.

### 2.3. Effect of Genotype, Multivariate Analysis

**Figure 4:**
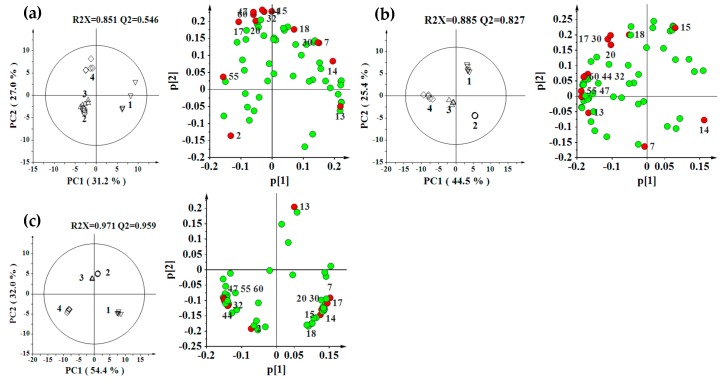
PCA scores plots and loading plots derived from LC-MS data for *S. miltiorrhiza* extracts from four different genotypes (1, Genotype 1; 2, Genotype 2; 3, Genotype 3; and 4, Genotype 4) for each location ((**a**) Zhuyang; (**b**) Changqing; and (**c**) Taian). 14 red colored metabolites in loading plots were the genotype-specific metabolites which we obtained from OPLS-DA: 2, Procatechuic acid; 7, Salvianolic acid F; 13, Salvianolic acid I; 14, Salvianolic acid E; 15, Rosmarinic acid; 17, Lithospermic acid; 18, Prolithospermic acid derivative; 20, Salvianolic acid B; 30, 7α-hydroxyallyl-royleanone; 32, Tanshinone IIB; 44, 15,16′-dihydrotanshinone I; 47, Methyltanshinonate; 55, 1,2′-dihydrotanshinone I; and 60, Tanshinone IIA.

In order to assess the effect of genotype, we subjected the data gathered from each location to PCA separately (Figure 4). The PCA scores plots displayed tight clustering for each group, and obvious separation for the *S. miltiorrhiza* grown from four genotypes with the first two principal components accounting for 58.2%–86.4% of variables. These results indicated the enormous intergroup differences in the metabolite contents for different genotypes.

The pairwise comparative OPLS-DA model showed enormous intergroup difference with the value of R^2^X and Q^2^ confirming the good model quality (Figure S2). Figure S2a shows OPLS-DA scores plots between Genotype 1 and Genotype 2 at three locations, respectively. Compared with Genotype 2, Genotype 1 had more phenolic acids (e.g., 7, 13, 14, 15, and 18) but less some other phenolic acids (e.g., 2, 17, and 20) at Zhuyang location (Figure S2a). This was consistent with the quantification in Table 2. At Changqing and Taian locations, compared with Genotype 2, Genotype 1 contained higher levels of phenolic acids (e.g., 15, 17, 18, and 20), 15,16′-dihydrotanshinone I (44) and tanshinone I (53) (Figure S2a), which was consistent with the results obtained from the absolute concentrations (Table 2). This result also showed that Genotype 1 samples had more phenolic acids than Genotype 2 samples at Changqing and Taian locations (Figure S2a).

Figure S2b shows OPLS-DA scores plots between Genotype 2 and Genotype 3 at each of the three locations. Genotype 3 contained more phenolic acids (e.g., 2, 7, 13, 15, 17, 18, and 20) and methyltanshinonate (47) than Genotype 2 at Zhuyang location (Figure S2b), which was consistent with the quantification (Table 2). Similarly, at Changqing and Taian locations, compared with Genotype 2, Genotype 3 had more lithospermic acid (17), salvianolic acid B (20), 7α-hydroxyallyl-royleanone (30), and tanshinones (e.g., 32, 44, 47, 52, 55, and 60) (Figure S2b). The conclusion was consistent with the absolute concentrations (Table 2). The above result showed that Genotype 3 had more phenolic acids and tanshinones than Genotype 2 across three locations.

Figure S2c shows OPLS-DA scores plots between Genotype 3 and Genotype 4 at three locations, respectively. Compared with Genotype 3, Genotype 4 contained more tanshinones (e.g., 44, 47, and 60) but less phenolic acids (e.g., 14, 15, and 17, and 20) at Zhuyang location (Figure S2c). This result was completely agreeable with the quantification (Table 2). Likewise, at Changqing and Taian locations, Genotype 3 showed higher levels of phenolic acids (e.g., 7, 17, and 20) but lower amounts of tanshinones (e.g., 44, 47, 52, 55, and 60) (Figure S2c), which broadly agreed with the absolute quantification (Table 2). The above results showed that Genotype 4 had more tanshinones while Genotype 3 had more phenolic acids across three locations.

Through the above research, the loadings plots revealed the 14 metabolites contributed significantly to the classifications of different genotypes, including 7α-hydroxyallyl-royleanone, procatechuic acid, salvianolic acid F, salvianolic acid I, salvianolic acid E, salvianolic acid B, rosmarinic acid, lithospermic acid, prolithospermic acid derivative, tanshinone IIB, tanshinone IIA, 15,16′-dihydrotanshinone I, methyltanshinonate, and 1,2′-dihydrotanshinone I (Tables S4 and S5). These metabolites could be used as potential genotype-specific markers in *S. miltiorrhiza*. Great differences of these genotype-specific metabolites were also observed by PCA loading plots (Figure 4).

The overall metabolite composition was separated by the genotypes in Figure 4, indicating that genetic differences were showed in metabolic differences. This may be attributed to differences in the activity of key enzymes, and differences in the regulation of metabolic pathways. Previous research showed that the higher rosmarinic acid accumulation observed in *S. miltiorrhiza* genotypes might be a result of the up-regulation of several co-expressed genes (e.g., *SmPAL*, *SmC4H*, *Sm4CL* and *SmHPPD*) in this metabolic pathway [21]. These intergroup differences probably resulted from acclimatization and selective breeding.

Targeted profiles of eight specific metabolites revealed a large variation between different genotypes (Table 2). Genotype 3 had relatively higher rosmarinic acid, lithospermic acid, and salvianolic acid B than Genotype 2 and Genotype 4, not counting the Genotype 1 (Table 2). Thus, we regarded Genotype 3 as a high-phenolics genotype. Four tanshinones (*i.e.*, 15,16′-dihydrotanshinone I, cryptotanshinone, tanshinone I, tanshinone II A) had a similar variation rule in our study: Genotype 4 had relatively higher tanshinones content than did the other genotypes (Table 2). Variation in these health-related metabolites may result from artificial breeding aimed at selecting for high-yielding or high-quality features (e.g., high phenolic acids, and high tanshinones features). Nowadays, metabolic engineering have become an effective tool to artificial selectively produce phenolic acids and other secondary metabolites in *S. miltiorrhiza* [9].

### 2.4. Effect of Genotype-by-Environment Interaction

In order to evaluate the contribution of genotype and environment to variation of secondary metabolites, unsupervised PCA was used for paired locations and genotypes (Figure S3) because the holistic PCA failed to simply classify samples according to cultivar or location as the description in previous research [22]. If the environment had a main effect, the PC1 would separate different locations regardless of genotypes. In contrast, if the genetic background had a main effect, samples from the same genotype would gather on the same side in horizontal axis regardless of locations [17].

Our research showed that both location and genotype affected the content of bioactive compositions (Table 3), which was consistent with the description in phenolic acid of olive oil [22]. The dominant factor for the variation of secondary metabolites was subject to change, but it was regular and predictable. The predominant factor was shown in Table 3 in different cases, which was a summary of Figure S3.

The environmental difference masked the genotypic difference when we compare Zhuyang location with two other locations (Table 3) because there was a significant metabonomic difference between Zhuyang and the two other locations (Figure 1). For example, in Figure S3a, an obvious separation based on the differentiation of locations (Zhuyang and Changqing) was observed on PC1 accounting for 47.7% of the variation, whereas a differentiation according to genotypes (Genotype 1 and Genotype 2) was obtained along PC2 explained 23.6% of the variation. Thus, the growing location had a greater impact on metabolite variations than genotype in this case. This finding agreed with Boulila *et al.* who reported that growing location had a greater impact on phenolic acid in *Marrubium vulgare* L. (Lamiaceae) [19].

Conversely, with the decrease of the environmental difference and the increase of the genotypic difference, the genetic background gradually played a leading role (Table 3). For example, in Figure S3c, PC1 explained 42.2% of the total variance, and distinguished the groups based on different genotypes (Genotype 1 and Genotype 4), while PC2, which explained 26.1% of the total variance, distinguished the groups in accordance with different locations (Changqing and Taian). This result suggested that the genetic difference was larger than the environmental difference in some cases. This finding agreed with Lee *et al.* who showed that genotype had a greater impact on terpenoids than growing location in *Curcuma* species [17]. Furthermore, cultivars also had a great effect for flavones content in *Ocimum* species [20]. 

## 3. Experimental Section

### 3.1. Field Experiment Design

The experiment presented here was carried out at Zhuyang (Shandong province, China; 36:17:48N; 117:21:41E), Changqing (Shandong province, China; 36:26:4N; 116:46:55E), and Taian (Shandong Agricultural University Experimental Farm, Taian, China; 36:9:50N; 117:9:47E) during 2014. Zhuyang location was a sandy loam (low-nutrient) sown with peanuts for >3 years. Analyses of this soil before planting showed about 5.1 g/kg, and 34.88 mg/kg in soil organic carbon, and available N. Changqing location was a loam sown with chrysanthemums for >1 years. Analyses of this soil before planting showed about 11.4 g/kg, and 40.03 mg/kg in soil organic carbon, and available N. Taian location was a loam with high-nutrient. Analyses of this soil showed about 15.4 g/kg, and 55.28 mg/kg in soil organic carbon, and available N. Soil organic carbon and available N were determined as described by Zhang *et al*. [32].

Simultaneously, four *S. miltiorrhiza* genotypes, namely, Genotype 1, Genotype 2, Genotype 3 and Genotype 4, which differed in disease resistance, productivity and morphological characters, were used (Figure S4, Table S3). These four genotypes were bred from naturally occurring populations on Tai Mountain through individual selection, and had stable agronomy characters over the years (Table S3). Their genetic linkage map had been constructed by our groups [33]. 

The fresh roots of four *S. miltiorrhiza* genotypes (Genotypes 1, 2, 3, and 4) were obtained from Zhuyang, Changqing, and Taian of Shandong province, China. The same *S. miltiorrhiza* genotype was reproduced by root segment. Each genotype was planted in five randomized blocks at every location. The treatment block was 10 m × 10 m in size. *S. miltiorrhiza* root segment was planted in ridges (distance between rows: 35 cm, distance between root segment within the row: 25 cm) at 10 April 2014. Then, these samples were cultivated under identical agronomic practice (e.g., irrigation, fertilization, and pesticide and herbicide applications) in different locations from April to 15 November 2014. The roots of *S. miltiorrhiza* with 1 cm diameter were collected from each genotype and block, air-dried at ambient with dark and good ventilation, and ground into power. Voucher specimens were preserved in the State Key Laboratory of Crop Biology, College of Agronomy, Shandong Agricultural University, Taian, China.

### 3.2. Extraction and Analysis of Secondary Metabolites

Secondary metabolites were extracted using the method described by Fang *et al.* [23]. Dried sample powder (50 mg) was mixed with 50 mL of extraction solvent (80% methanol and 20% water, *v/v*). Then, the extracts were sonicated under strictly controlled temperature for 40 min, and filtered through a microfiltration membrane (0.22 μm). Five biological replicates were prepared for each genotype and location, resulting in a total of 60 extracts. Quality control samples were also prepared by pooling material of several randomly chosen samples to check the technical variation. The quality control samples were injected after every 10 sample extracts.

Analyses were carried out on a high pressure liquid chromatography (HPLC) combined with a Finnigan LCQ-Deca XP Max mass spectrometer (Thermo Fisher Scientific West Palm Beach, FL, USA). The Finnigan LC system consisted of an autosampler, a pump and a photodiode array detector. We separated the extracts via a Hypersil GOLD C18 column (2.1 × 150 mm, 3 μm). Acetonitrile (A) and water (B), both acidified with 0.1% acetic acid (*v/v*), were the mobile phase components. A linear gradient was used as follows: 0–30 min, 15%–30% A; 30–35 min, 30%–50% A; 35–40 min, 50%–60% A; 40–70 min, 60%–80% A [23]. Injection volume was set at 10 μL for each sample. The flow rate was 0.2 mL/min. The optimized parameters for electrospray ionization were set as follows: gas temperature was set at 180 °C; nebulizer gas pressure was set at 80,000 Pa; the source voltage was set at 4500 V (negative mode) and −4000 V (positive mode). The spectral range was 50–900 (*m/z*) and the monitoring wavelength was 280 nm. Finally, we used the Xcalibur version 2.1.0 software (Thermo Fisher Scientific, Waltham, MA, USA) for data analysis.

The chromatographic condition and instrumentation of HPLC for quantitative determination were the same as described above, except for replacing acetic acid by phosphoric acid (0.01%). Standard substances of danshensu, rosmarinic acid, lithospermic acid, salvianolic acid B, 15,16′-dihydrotanshinone I, cryptotanshinone, tanshinone I, and tanshinone IIA were obtained from the National Institute for the Control of Pharmaceuticals and Biological Products (Beijing, China). Quantitative determination of the eight bioactive compositions was performed using external standards by means of a six points calibration curve. For LC-UV date, the peaks from a fixed monitoring wavelength (280 nm) were employed for statistics analysis.

### 3.3. Multivariate Data Analysis

The peak integrals from the LC-UV chromatograms obtained from the Xcalibur version 2.1.0 software were used for PCA and OPLS-DA analyses. PCA and OPLS-DA were carried out using SIMCA-P^+^ 13.0.2 software (Umetrics, Sweden) [25]. PCA analyses were performed with mean center scaling, and showed in the form of the scores plot and loading plot. OPLS-DA results were carried out with unit variance scaling, conducted regarding the class information as Y variables, and showed in the form of scores plot and coefficient-coded loading plot. The OPLS-DA loading plot was carried out by using MATLAB 7.0 scripts (http://www.mathworks.com/), and color-coded with the correlation coefficient [25]. The hot colored (red and orange) variables represented significant contribution to the classification than the cold (blue and green) colored ones. The quality of the model was estimated by the parameter of the cross validation (5-fold): R^2^X, which showed the total explained variables for all LC-MS data, and Q^2^ which showed the predictability of the model. The coefficient cutoff value of 0.90 was used according to the statistical significance (*p* < 0.001). 

### 3.4. Analysis of Enzyme Activities

The fresh roots (0.8 g) of *S. miltiorrhiza* were homogenized with 6 mL of 0.05 mol/L Tris-HCl buffer (pH 8.9) at 4 °C. The Tris-HCl buffer contained 15 mmol/L of β-mercaptoethanol, 5 mmol/L of ethylene diamine tetraacetic acid (EDTA), 5 mmol/L of Vitamin C (Vc), 10% of glycerol (*v/v*), 0.15% of polyvinyl pyrrolidone (PVP, *w/v*) and 0.017% of phenylmethanesulfonyl fluoride (PMSF, *w/v*). The homogenate was sonicated for 2 min and centrifuged at 12,000× *g* (20 min, 4 °C) to make sure the separation of the supernatant. The extractions were performed in triplicate.

The activity of phenylalanine ammonia-lyase (PAL, EC 4.3.1.24) and cinnamyl alcohol-NADPH dehydrogenase (CAD, EC 1.1.1.2) were determined using the method of Morrison *et al*. [34]. The assay for tyrosine ammonic-lyase (TAL, EC 4.3.1.23) activity was conducted using the same protocol as that for PAL except l-tyrosine was added to the tyrosine substrate [35]. The analysis of 4-coumarate-CoA ligase (4CL, EC 6.2.1.12) activity followed the method reported by Knobloch *et al*. [36]. We calculated all the results based on total protein content. Total protein content was measured by the Bradford method [37]. 

For the enzyme activities and absolute concentration of eight metabolites, one way-ANOVA according to Bonferroni correction was carried out by using SPSS 20 software to evaluate whether difference was significant. We also applied Bonferroni correction for a *p*-value of 0.000757 (0.05/66) to account for multiple comparisons of the relative concentration.

## 4. Conclusions

In conclusion, the *S. miltiorrhiza* genotypes grown in Zhuyang as compared to those grown in Changqing and Taian had lower yield and higher phenolic acids, tanshinones content, PAL, 4CL, CAD, and TAL activities. Furthermore, Genotype 1 could maintain relative salvianolic acid B stability, indicating that Genotype 1 was possibly related to resistances. Therefore, the article provided guidance for the selecting of locations and genotypes in *S. miltiorrhiza* cultivation and production. The contents of phenolic acids and tanshinones within *S. miltiorrhiza* were affected by locations as well as genotypes. Therefore, the efficacy of *S. miltiorrhiza* might vary with different locations and genotypes. The high concentrations of secondary metabolites might improve their therapeutic efficacies, and could be enhanced by optimizing locations and genotypes. Further clinical research is warranted to consider in future studies. OPLS-DA showed that phenolic acids and tanshinones were important markers for characterizing different genotypes and locations. It is concluded that LC/MS-based metabolomics was a holistic and effective method to monitor variations of the bioactive composition in herbal medicines resulting from genotypes and environmental factors.

## Figures and Tables

**Table 1:** Annotated compounds in *S. miltiorrhiza* samples based on literature searches.

Peak No.	Molecular Weight	λ_max_ (nm)	RT (min)	Putative Annotation	Negative Ion Mode		Positive Ion Mode			Ref.	Annot. Level ^§^
					[M − H]^−^	Major Fragments	[M + Na]^+^	[M + H]^+^	Major Fragments		
1	198	282(sh)–326	3.9	Danshensu	197	395, 257, 179, 135	221	199	181, 163, 153	^abcd^	*1*
2	154	228–261–280	4.7	Procatechuic acid	153	213, 307,109	- ^†^	-	-	^be^	*2*
4	138	232–281–313	6.6	Procatechuic aldehyde	137	275	161	-	-	^be^	*2*
5	168	219–294–330	7.6	Vanillic acid	167	-	-	-	-	^b^	*2*
6	180	240–281–326	8.2	Caffeic acid	179	359, 239, 161, 151, 135	-	181	163	^bce^	*2*
7	314	275–318	12.4	Salvianolic acid F	313	269, 159, 109	-	-	-	^bd^	*2*
10	194	290(sh)–323	18.5	Ferulic acid	193	175, 147	-	195	177, 163	^bce^	*2*
13	538	288	20.0	Salvianolic acid I	537	559, 493, 339, 295	561	539	521, 323	^bcd^	*2*
14	718	214–299(sh)–329	22.1	Salvianolic acid E	717	519	741	-	-	^b^	*2*
15	360	331(sh)	23.1	Rosmarinic acid	359	719, 419, 341, 315, 197, 179, 161	383	361	181, 163	^abcde^	*1*
16	286	259–299–320–373	23.6	Luteolin	285	133	-	-	-	^ND^	*2*
17	538	255–308	24.3	Lithospermic acid	537	493, 449, 339, 313, 295	561	539	521, 493, 341	^abcd^	*1*
18	670	260–280	26.0	Prolithospermic acid derivative	669	551, 519	693	671	521, 433, 373	^cd^	*2*
20	718	289–310	27.2	Salvianolic acid B	717	339, 321, 295, 279, 277	741	719	521	^abce^	*1*
21	718	225(sh)–285–305	27.6	Isosalvianolic acid B	717	519	741	-	-	^bc^	*2*
23	718	225(sh)–285–305	30.0	Salvianolic acid L	717	519, 501, 339, 295, 197	741	719	521, 323, 295	^bcd^	*2*
24	494	225–285–310	31.1	Salvianolic acid A	493	295	-	-	-	^abe^	*2*
26	312	226–265–310	34.2	Tanshindiol C	-	-	-	313	373, 295, 267	^b^	*2*
29	344	260–340(sh)	38.7	Royleanone-4	-	-	367	345	327, 309	^b^	*2*
30	312	310–340(sh)	39.6	7α-hydroxyallyl-royleanone	-	-	353	331	313	^b^	*2*
32	310	275(sh)–254	43.5	Tanshinone II B	-	-	-	311	293, 283, 265, 251	^bcd^	*2*
33	310	225–265	44.0	1-ketoisocryptotanshinone	-	-	333	311	293	^cd^	*2*
34 ^‡^	488	225–275–325	45.3	Tormentic acid	487	470, 469, 467, 423	-	-	-	^cd^	*2*
36	340	275(sh)–380	45.4	Trijuganone C	-	-	363	341	309, 281, 273	^bcd^	*2*
37	296	230–260–330	45.9	Danshenxinkun A	295	265	-	-	-	^c^	*2*
39	414	230(sh)–255–330	46.1	β-sitosterol	-	-	437	415	-	^cd^	*2*
40	308	235(sh)–380–395	46.4	Tanshinaldehyde	-	-	331	309	-	^cd^	*2*
42	310	240(sh)–270	47.1	Przewa tanshinone A	-	-	-	311	293, 275, 247	^b^	*2*
44	278	245(sh)–285–330	47.8	15,16′-dihydrotanshinone I	-	-	301	279	261, 237, 233, 209	^abce^	*1*
46	314	250(sh)–380	48.7	Neocryptotanshinone	313	-	-	315	295, 279	^bc^	*2*
47	338	255(sh)–275	49.0	Methyltanshinonate	-	-	-	339	307, 297, 279, 278, 261	^b^	*2*
48	280	274(sh)–320	49.3	Trijuganone B	-	-	303	281	263, 235	^cd^	*2*
49	294	250(sh)	49.7	Trijuganone A	-	-	317	295	280	^b^	*2*
50	300	255	50.1	Miltipolone	299	-	-	-	-	^cd^	*2*
52	296	265(sh)–355	51.7	Cryptotanshinone	-	-	319	297	615, 279, 251, 237	^abcde^	*1*
53	276	250(sh)	53.6	Tanshinone I	-	-	299	277	575, 259, 249, 231	^abcde^	*1*
55	278	210–230–290(sh)	56.0	1,2′-dihydrotanshinone I	-	-	301	279	339, 261, 233	^cd^	*2*
59	280	245(sh)–275	58.1	Dehydromiltirone	-	-	-	281	461, 266	^b^	*2*
60	294	270(sh)	58.5	Tanshinone IIA	-	-	317	295	611, 280, 262, 249	^abcde^	*1*
62	282	255(sh)–285	59.72	Miltirone	-	-	305	283	587, 268, 240	^bcd^	*2*

*S. miltiorrhiza* metabolites had been obtained from the followed literature records: ^a^ Ref. [23]; ^b^ Ref. [26]; ^c^ Ref. [25]; ^d^ Ref. [24]; ^e^ and Ref. [8]. ^†^, -, not determine. ^‡^ The relative concentrations of peak 34, 37, and 50 were not exact due to overlapping. ^§^ Annotation level was classified as recommended by the metabolomics standards initiative: *1* identified metabolites and *2* putatively annotated compounds.

**Table 2:** Metabolites content of *S. miltiorrhiza* extracts from different genotypes for each location (Zhuyang, Changqing, Taian).

Putative Annotation	Genotype 1	Genotype 2	Genotype 3	Genotype 4
**(a) Zhuyang, g/kg DW**				
Danshensu	0.25 ± 0.02 ^a^	0.14 ± 0.01 ^c^	0.21 ± 0.01 ^b^	0.23 ± 0.01 ^b^
Rosmarinic acid	2.38 ± 0.17 ^c^	1.90 ± 0.06 ^d^	3.98 ± 0.23 ^a^	3.65 ± 0.14 ^b^
Lithospermic acid	1.71 ± 0.08 ^c^	2.04 ± 0.09 ^b^	2.82 ± 0.14 ^a^	2.74 ± 0.11 ^a^
Salvianolic acid B	39.23 ± 1.33 ^c^	40.73 ± 2.46 ^c^	54.08 ± 2.24 ^a^	45.78 ± 3.55 ^b^
15,16′-dihydrotanshinone I	1.14 ± 0.02 ^c^	0.94 ± 0.07 ^d^	1.33 ± 0.03 ^b^	2.19 ± 0.02 ^a^
Cryptotanshinone	3.65 ± 0.20 ^c^	5.02 ± 0.10 ^b^	5.21 ± 0.20 ^b^	7.95 ± 0.10 ^a^
Tanshinone I	1.79 ± 0.05 ^d^	1.99 ± 0.07 ^c^	2.43 ± 0.04 ^b^	3.62 ± 0.06 ^a^
Tanshinone IIA	3.94 ± 0.20 ^c^	5.52 ± 0.10 ^b^	5.33 ± 0.05 ^b^	6.65 ± 0.15 ^a^
**(b) Changqing, g/kg DW**				
Danshensu	0.18 ± 0.02	0.16 ± 0.03	0.15 ± 0.01	0.15 ± 0.01
Rosmarinic acid	2.51 ± 0.04 ^a^	1.51 ± 0.16 ^c^	2.03 ± 0.15 ^b^	1.53 ± 0.01 ^c^
Lithospermic acid	1.44 ± 0.09 ^b^	1.11 ± 0.14 ^c^	1.73 ± 0.07 ^a^	1.23 ± 0.11 ^c^
Salvianolic acid B	40.42 ± 3.54 ^b^	27.16 ± 1.03 ^d^	48.61 ± 1.65 ^a^	35.36 ± 2.76 ^c^
15,16′-dihydrotanshinone I	0.29 ± 0.01 ^b^	0.14 ± 0.01 ^c^	0.27 ± 0.03 ^b^	0.45 ± 0.03 ^a^
Cryptotanshinone	0.71 ± 0.04 ^c^	0.59 ± 0.03 ^d^	0.98 ± 0.01 ^b^	1.41 ± 0.03 ^a^
Tanshinone I	0.43 ± 0.04 ^c^	0.37 ± 0.03 ^c^	0.57 ± 0.04 ^b^	0.89 ± 0.03 ^a^
Tanshinone IIA	1.28 ± 0.10 ^c^	0.88 ± 0.03 ^d^	1.63 ± 0.05 ^b^	2.02 ± 0.20 ^a^
**(c) Taian, g/kg DW**				
Danshensu	0.33 ± 0.02 ^a^	0.14 ± 0.03 ^c^	0.15 ± 0.02 ^c^	0.21 ± 0.02 ^b^
Rosmarinic acid	5.34 ± 0.24 ^a^	1.22 ± 0.01 ^c^	1.62 ± 0.03 ^b^	1.35 ± 0.13 ^c^
Lithospermic acid	2.96 ± 0.22 ^a^	0.95 ± 0.05 ^a^	1.41 ± 0.24 ^b^	0.83 ± 0.08 ^c^
Salvianolic acid B	49.13 ± 0.85 ^a^	24.27 ± 0.88 ^a^	28.96 ± 1.16 ^c^	23.82 ± 0.97 ^c^
15,16′-dihydrotanshinone I	0.13 ± 0.03 ^c^	0.07 ± 0.02 ^d^	0.20 ± 0.02 ^b^	0.37 ± 0.01 ^a^
Cryptotanshinone	0.17 ± 0.01 ^c^	0.16 ± 0.03 ^c^	0.24 ± 0.02 ^b^	1.48 ± 0.05 ^a^
Tanshinone I	0.25 ± 0.05 ^b^	0.31 ± 0.06 ^b^	0.31 ± 0.02 ^b^	0.70 ± 0.03 ^a^
Tanshinone IIA	0.19 ± 0.10 ^c^	0.22 ± 0.07 ^c^	0.47 ± 0.05 ^b^	2.13 ± 0.02 ^a^

Values in each row having different lowercase letters (a, b, c, and d) are significantly different at *p* < 0.001/6 (Bonferroni correction).

**Table 3:** The predominant factor for the variation of chemical compositions obtained from paired locations and genotypes (ZY, Zhuyang; CQ, Changqing; TA, Taian. This table was a summary of Figure S3).

Paired Genotypes	ZY *vs.* CQ	ZY *vs.* TA	CQ *vs.* TA
Genotype 1 *vs.* Genotype 2	location ^a^	location	location
Genotype 1 *vs.* Genotype 3	location	location	location
Genotype 1 *vs.* Genotype 4	location	location	genotype ^b^
Genotype 2 *vs.* Genotype 3	location	location	genotype
Genotype 2 *vs.* Genotype 4	location	location	genotype
Genotype 3 *vs.* Genotype 4	location	location	location

^a^ “location” denoted that the growing location had a greater effect on metabolite variation than genetic background. ^b^ “genotype” denoted that the genetic background had a greater effect on metabolite variation than locations.

## Supplementary Materials

Supplementary materials can be accessed at: http://www.mdpi.com/1420-3049/21/4/414/s1.
