# Supplementary material for: Effect of Genotype and Environment on Salvia miltiorrhiza Roots Using LC/MS-Based Metabolomics
Source: Molecules. 2016 Mar 26;21(4):414. doi: 10.3390/molecules21040414 (PMC6273704; doi:10.3390/molecules21040414)
Supplement: Supplementary file 1 [file molecules-21-00414-s001.pdf]

Effect of Genotype and Environment on *Salvia miltiorrhiza* Roots Using LC/MS–Based Metabolomics

Qi Zhao, Zhenqiao Song, Xinsheng Fang, Yuling Pan, Linlin Guo, Tian Liu and Jianhua Wang

Table S1. The correlation coefficients from OPLS-DA of *S. miltiorrhiza* extracts from paired locations for each genotype (ZY, Zhuyang; CQ, Changqing; TA, Taian).

Putative Annotation	Genotype 1			Genotype 2			Genotype 3			Genotype 4		
	ZY/CQ	ZY/TA	CQ/TA	ZY/CQ	ZY/TA	CQ/TA	ZY/CQ	ZY/TA	CQ/TA	ZY/CQ	ZY/TA	CQ/TA
Salvianolic acid F	**−0.998** [a]	**0.991**	**1.000**	0.365	0.369	−0.111	0.830	**−0.987**	**−0.995**	**−0.997**	**−0.999**	**−0.911**
Salvianolic acid I	**−0.998**	**−0.999**	**0.987**	**−1.000**	**−0.984**	**0.985**	**−0.990**	**−0.999**	**−0.971**	0.149	**−0.998**	**−0.999**
Salvianolic acid E	**−0.999**	**−0.998**	**0.996**	**−0.993**	**−0.994**	**−0.964**	**−1.000**	**−1.000**	**−1.000**	**−0.991**	**−0.991**	**−0.985**
Rosmarinic acid	0.790	**0.999**	**0.997**	**−0.979**	**−0.910**	−0.866	−0.816	**−0.983**	**−0.975**	**−0.999**	**−0.984**	−0.826
Lithospermic acid	**−0.967**	**0.987**	**0.989**	**−0.999**	**−0.999**	**−0.979**	−0.864	**−0.998**	**−0.976**	**−0.999**	**−1.000**	**−0.992**
Prolithospermic acid derivative	**−0.956**	**0.998**	**0.995**	**−0.999**	**−0.998**	**−0.980**	**0.956**	**−0.999**	**−0.992**	−0.494	−0.438	0.749
Salvianolic acid B	**0.946**	**0.975**	**0.966**	**−0.998**	**−0.998**	−0.884	**−0.987**	**−0.983**	**−0.999**	**−0.960**	**−0.998**	**−0.960**
Tanshinone IIB	**−0.931**	**−0.941**	**−0.980**	**−0.962**	**−0.967**	**−0.994**	**−0.926**	**−0.932**	0.342	**−0.963**	**−0.974**	**−0.924**
Trijuganone C	**−0.915**	**−0.923**	−0.658	**−0.978**	**−0.978**	−0.867	**−0.997**	**−0.999**	**−0.955**	−0.799	**−0.939**	**−0.996**
15,16′-dihydrotanshinone I	−0.872	**−0.901**	**−0.971**	**−0.997**	**−0.997**	**−0.994**	**−0.961**	**−0.971**	**−0.949**	**−0.994**	**−0.998**	**−0.997**
Methyltanshinonate	**−0.924**	**−0.942**	**−0.995**	**−1.000**	**−0.999**	**−0.999**	**−0.998**	**−0.999**	**−0.986**	**−0.998**	**−0.997**	**−0.998**
Trijuganone B	−0.420	**−0.919**	−0.627	**−0.988**	**−0.991**	**−0.999**	**−0.965**	**−0.973**	**−0.996**	**−0.988**	**−0.990**	−0.069
Cryptotanshinone	−0.886	**−0.928**	**−0.942**	**−0.978**	**−0.981**	**−1.000**	**−0.962**	**−0.967**	**−0.953**	**−0.951**	**−0.936**	0.841
Tanshinone I	−0.806	−0.845	−0.811	**−0.992**	**−0.992**	**1.000**	−0.839	**−0.906**	**−0.981**	**−0.998**	**−0.998**	**−0.996**
1,2′-dihydrotanshinone I	−0.594	−0.769	**−0.997**	**−1.000**	**−0.999**	**−0.996**	**−0.998**	**−1.000**	**−0.995**	**−0.993**	**−0.990**	−0.740
Tanshinone IIA	−0.860	**−0.922**	**−0.997**	**−1.000**	**−1.000**	**−0.996**	**−0.997**	**−0.999**	**−0.991**	**−0.993**	**−0.990**	−0.226

[a] Positive and negative signs indicate positive and negative correlation in the concentration, respectively. The coefficient of 0.90 was used as the cutoff value based on the significant difference evaluation ($p < 0.001$).

Table S2. *p*-Value of *S. miltiorrhiza* extracts from paired locations for each genotype (ZY, Zhuyang; CQ, Changqing; TA, Taian).

Putative Annotation	Genotype 1			Genotype 2			Genotype 3			Genotype 4		
	ZY/CQ	ZY/TA	CQ/TA	ZY/CQ	ZY/TA	CQ/TA	ZY/CQ	ZY/TA	CQ/TA	ZY/CQ	ZY/TA	CQ/TA
Salvianolic acid F	$\mathbf{1.4 \times 10^{-17}}$ [a],*	$\mathbf{5.0 \times 10^{-11}}$ *	$\mathbf{7.4 \times 10^{-19}}$ *	5.5×10^{-1}	7.7×10^{-1}	1.1×10^{-1}	7.6×10^{-4}	$\mathbf{2.4 \times 10^{-9}}$ *	$\mathbf{1.1 \times 10^{-10}}$ *	$\mathbf{3.7 \times 10^{-14}}$ *	$\mathbf{5.7 \times 10^{-15}}$ *	$\mathbf{2.3 \times 10^{-5}}$ *
Salvianolic acid I	$\mathbf{7.5 \times 10^{-22}}$ *	$\mathbf{1.4 \times 10^{-21}}$ *	$\mathbf{8.8 \times 10^{-7}}$ *	$\mathbf{1.2 \times 10^{-13}}$ *	$\mathbf{5.8 \times 10^{-10}}$ *	$\mathbf{3.3 \times 10^{-10}}$ *	$\mathbf{3.2 \times 10^{-9}}$ *	$\mathbf{2.4 \times 10^{-13}}$ *	$\mathbf{7.3 \times 10^{-8}}$ *	2.6×10^{-1}	$\mathbf{2.6 \times 10^{-14}}$ *	$\mathbf{2.3 \times 10^{-14}}$ *
Salvianolic acid E	$\mathbf{1.1 \times 10^{-18}}$ *	$\mathbf{2.2 \times 10^{-16}}$ *	$\mathbf{2.2 \times 10^{-13}}$ *	$\mathbf{5.6 \times 10^{-12}}$ *	$\mathbf{1.1 \times 10^{-12}}$ *	$\mathbf{1.8 \times 10^{-4}}$ *	$\mathbf{1.5 \times 10^{-17}}$ *	$\mathbf{2.3 \times 10^{-20}}$ *	$\mathbf{8.1 \times 10^{-16}}$ *	$\mathbf{7.1 \times 10^{-11}}$ *	$\mathbf{2.8 \times 10^{-11}}$ *	$\mathbf{2.5 \times 10^{-4}}$ *
Rosmarinic acid	1.2×10^{-2}	$\mathbf{8.1 \times 10^{-15}}$ *	$\mathbf{1.9 \times 10^{-14}}$ *	$\mathbf{2.7 \times 10^{-7}}$ *	$\mathbf{3.1 \times 10^{-4}}$ *	$\mathbf{3.1 \times 10^{-4}}$ *	5.1×10^{-3}	$\mathbf{1.4 \times 10^{-8}}$ *	$\mathbf{4.5 \times 10^{-7}}$ *	$\mathbf{5.0 \times 10^{-11}}$ *	$\mathbf{7.6 \times 10^{-10}}$ *	9.5×10^{-4}
Lithospermic acid	$\mathbf{8.7 \times 10^{-5}}$ *	$\mathbf{4.0 \times 10^{-10}}$ *	$\mathbf{5.4 \times 10^{-11}}$ *	$\mathbf{2.4 \times 10^{-17}}$ *	$\mathbf{3.0 \times 10^{-18}}$ *	$\mathbf{8.8 \times 10^{-9}}$ *	$\mathbf{2.6 \times 10^{-4}}$ *	$\mathbf{5.5 \times 10^{-10}}$ *	$\mathbf{3.1 \times 10^{-8}}$ *	$\mathbf{5.7 \times 10^{-18}}$ *	$\mathbf{3.6 \times 10^{-19}}$ *	$\mathbf{5.3 \times 10^{-11}}$ *
Prolithospermic acid derivative	$\mathbf{2.5 \times 10^{-6}}$ *	$\mathbf{5.3 \times 10^{-12}}$ *	$\mathbf{2.0 \times 10^{-13}}$ *	$\mathbf{2.7 \times 10^{-13}}$ *	$\mathbf{3.4 \times 10^{-15}}$ *	$\mathbf{3.8 \times 10^{-9}}$ *	$\mathbf{2.3 \times 10^{-7}}$ *	$\mathbf{1.7 \times 10^{-8}}$ *	$\mathbf{1.9 \times 10^{-11}}$ *	2.5×10^{-1}	3.6×10^{-1}	2.6×10^{-1}
Salvianolic acid B	$\mathbf{1.1 \times 10^{-4}}$ *	$\mathbf{1.6 \times 10^{-8}}$ *	$\mathbf{1.1 \times 10^{-7}}$ *	$\mathbf{2.0 \times 10^{-12}}$ *	$\mathbf{2.2 \times 10^{-13}}$ *	$\mathbf{8.9 \times 10^{-5}}$ *	$\mathbf{6.8 \times 10^{-11}}$ *	$\mathbf{2.9 \times 10^{-9}}$ *	$\mathbf{1.1 \times 10^{-13}}$ *	$\mathbf{5.2 \times 10^{-7}}$ *	$\mathbf{8.7 \times 10^{-11}}$ *	$\mathbf{1.2 \times 10^{-7}}$ *
Tanshinone IIB	$\mathbf{4.2 \times 10^{-6}}$ *	$\mathbf{1.7 \times 10^{-6}}$ *	$\mathbf{1.1 \times 10^{-5}}$ *	$\mathbf{1.2 \times 10^{-7}}$ *	$\mathbf{7.0 \times 10^{-8}}$ *	$\mathbf{1.9 \times 10^{-4}}$ *	$\mathbf{4.4 \times 10^{-6}}$ *	$\mathbf{4.6 \times 10^{-6}}$ *	7.3×10^{-1}	$\mathbf{7.3 \times 10^{-8}}$ *	$\mathbf{2.9 \times 10^{-8}}$ *	$\mathbf{8.7 \times 10^{-5}}$ *
Trijuganone C	$\mathbf{1.1 \times 10^{-5}}$ *	$\mathbf{7.2 \times 10^{-6}}$ *	1.2×10^{-1}	$\mathbf{3.4 \times 10^{-9}}$ *	$\mathbf{2.5 \times 10^{-9}}$ *	1.1×10^{-1}	$\mathbf{4.9 \times 10^{-14}}$ *	$\mathbf{4.1 \times 10^{-14}}$ *	$\mathbf{1.2 \times 10^{-6}}$ *	1.8×10^{-3}	$\mathbf{3.2 \times 10^{-6}}$ *	$\mathbf{2.6 \times 10^{-4}}$ *
15,16′-dihydrotanshinone I	$\mathbf{1.9 \times 10^{-4}}$ *	$\mathbf{4.3 \times 10^{-5}}$ *	$\mathbf{9.6 \times 10^{-5}}$ *	$\mathbf{1.3 \times 10^{-14}}$ *	$\mathbf{7.9 \times 10^{-15}}$ *	$\mathbf{1.3 \times 10^{-4}}$ *	$\mathbf{1.6 \times 10^{-7}}$ *	$\mathbf{4.7 \times 10^{-8}}$ *	$\mathbf{6.3 \times 10^{-4}}$ *	$\mathbf{5.2 \times 10^{-13}}$ *	$\mathbf{6.8 \times 10^{-14}}$ *	$\mathbf{8.0 \times 10^{-5}}$ *
Methyltanshinonate	$\mathbf{9.7 \times 10^{-6}}$ *	$\mathbf{1.8 \times 10^{-6}}$ *	$\mathbf{5.5 \times 10^{-4}}$ *	$\mathbf{5.9 \times 10^{-20}}$ *	$\mathbf{2.0 \times 10^{-20}}$ *	$\mathbf{8.7 \times 10^{-8}}$ *	$\mathbf{9.7 \times 10^{-17}}$ *	$\mathbf{8.7 \times 10^{-18}}$ *	$\mathbf{5.1 \times 10^{-9}}$ *	$\mathbf{1.6 \times 10^{-13}}$ *	$\mathbf{4.9 \times 10^{-15}}$ *	$\mathbf{4.9 \times 10^{-8}}$ *
Trijuganone B	4.0×10^{-1}	$\mathbf{5.1 \times 10^{-4}}$ *	9.8×10^{-2}	$\mathbf{1.0 \times 10^{-10}}$ *	$\mathbf{3.1 \times 10^{-11}}$ *	$\mathbf{8.3 \times 10^{-5}}$ *	$\mathbf{7.5 \times 10^{-8}}$ *	$\mathbf{2.9 \times 10^{-8}}$ *	$\mathbf{8.5 \times 10^{-5}}$ *	$\mathbf{3.2 \times 10^{-10}}$ *	$\mathbf{3.0 \times 10^{-10}}$ *	1.7×10^{-1}
Cryptotanshinone	$\mathbf{6.0 \times 10^{-5}}$ *	$\mathbf{5.2 \times 10^{-6}}$ *	$\mathbf{2.6 \times 10^{-4}}$ *	$\mathbf{8.2 \times 10^{-9}}$ *	$\mathbf{2.2 \times 10^{-9}}$ *	$\mathbf{2.6 \times 10^{-4}}$ *	$\mathbf{1.5 \times 10^{-7}}$ *	$\mathbf{4.2 \times 10^{-8}}$ *	$\mathbf{5.3 \times 10^{-4}}$ *	$\mathbf{8.9 \times 10^{-7}}$ *	$\mathbf{1.8 \times 10^{-6}}$ *	1.5×10^{-1}
Tanshinone I	1.1×10^{-3}	$\mathbf{3.6 \times 10^{-4}}$ *	1.1×10^{-1}	$\mathbf{7.8 \times 10^{-12}}$ *	$\mathbf{1.1 \times 10^{-11}}$ *	$\mathbf{2.7 \times 10^{-4}}$ *	$\mathbf{2.6 \times 10^{-4}}$ *	$\mathbf{2.5 \times 10^{-5}}$ *	$\mathbf{4.1 \times 10^{-4}}$ *	$\mathbf{1.9 \times 10^{-14}}$ *	$\mathbf{1.1 \times 10^{-15}}$ *	$\mathbf{7.0 \times 10^{-8}}$ *
1,2′-dihydrotanshinone I	5.8×10^{-2}	2.9×10^{-3}	$\mathbf{3.9 \times 10^{-4}}$ *	$\mathbf{2.3 \times 10^{-18}}$ *	$\mathbf{5.8 \times 10^{-19}}$ *	$\mathbf{1.2 \times 10^{-7}}$ *	$\mathbf{3.7 \times 10^{-17}}$ *	$\mathbf{7.3 \times 10^{-19}}$ *	$\mathbf{2.9 \times 10^{-12}}$ *	$\mathbf{9.8 \times 10^{-11}}$ *	$\mathbf{3.4 \times 10^{-11}}$ *	1.7×10^{-1}
Tanshinone IIA	$\mathbf{2.2 \times 10^{-4}}$ *	$\mathbf{8.2 \times 10^{-6}}$ *	$\mathbf{1.0 \times 10^{-4}}$ *	$\mathbf{2.6 \times 10^{-18}}$ *	$\mathbf{4.8 \times 10^{-19}}$ *	$\mathbf{1.2 \times 10^{-8}}$ *	$\mathbf{4.6 \times 10^{-15}}$ *	$\mathbf{1.2 \times 10^{-16}}$ *	$\mathbf{9.0 \times 10^{-10}}$ *	$\mathbf{6.7 \times 10^{-11}}$ *	$\mathbf{5.5 \times 10^{-11}}$ *	1.5×10^{-1}

[a] Multiple testing was corrected by Bonferroni correction. *$p < 7.57 \times 10^{-4}$ (0.05/66).

Table S3. Morphological characters and disease resistance of four genotypes.

Morphological characters and disease resistance	Genotype 1	Genotype 2	Genotype 3	Genotype 4	Significance
Cultivar or strain	cultivar	strain	cultivar	strain	
Accession name	Shandong Danshen NO.1	-	Shandong Danshen NO.2	-	
Leaf color	dark green	light green	dark green	light green	
Cauline basal petiole color	purple	purple	purple	green	
Plant types	erect type	spreading type	erect type	central type	
Average blade length (cm)	9 a	6 b	5 b	6 b	***
Average width of blade (cm)	7 a	3 c	4 b	5 b	**
Average plant height (cm)	51 a	40 b	44 b	63 a	*
Average crown width (cm^2)	2400 a	2000 b	1600 c	1700 c	***
Largest stem diameter (mm)	11 a	6 b	3 c	7 b	***
Cauline basal ramification number	8	6	10	7	NS
Longest root length (cm)	29 a	27 b	26 b	16 c	**
Largest root diameter (mm)	10 b	13 a	8 c	5 d	**
Average number of roots [a]	14 c	17 b	6 d	20 a	**
Root dry matter ($g.plant^{-1}$)	275 a	298 a	84 b	69 b	***
Root rot	2% b	4% a	4% a	4% a	**
Root knot nematode	2% c	7% a	6% b	5% b	**

[a] Diameter > 2mm. [b] Values in each row having different lowercase letters (a, b, c, and d) were significantly different at $p < 0.05$. NS, not significant ($p > 0.05$); * $p < 0.05$; ** $p < 0.01$; *** $p < 0.001$.

Table S4. The correlation coefficients from OPLS–DA of *S. miltiorrhiza* extracts from different genotypes for each locations (1, Genotype 1; 2, Genotype 2; 3, Genotype 3; 4, Genotype 4).

**Putative Annotation**	**Zhuyang**			**Changiqng**			**Taian**		
	**1/2**	**2/3**	**3/4**	**1/2**	**2/3**	**3/4**	**1/2**	**2/3**	**3/4**
Procatechuic acid	**0.997** [a]	**0.955**	**−0.990**	–	–	–	**−0.984**	**−0.985**	**0.997**
Salvianolic acid F	**−0.991**	**0.951**	**0.954**	**0.993**	**0.987**	**−0.992**	**−0.999**	**−0.973**	**−0.977**
Salvianolic acid I	**−0.998**	**0.996**	**−0.983**	–	**0.998**	0.529	**0.936**	0.865	**−0.999**
Salvianolic acid E	**−0.989**	−0.748	**−0.933**	**0.940**	**0.990**	**−0.996**	**−0.995**	**−0.997**	**0.996**
Rosmarinic acid	**−0.997**	**0.932**	**−0.994**	**−0.986**	**0.978**	**−0.983**	**−0.999**	0.642	−0.733
Lithospermic acid	**0.989**	**0.975**	**−0.997**	**−0.987**	**0.993**	**−0.988**	**−0.994**	**0.999**	**−0.997**
Prolithospermic acid derivative	**−0.997**	**0.992**	−0.618	**−0.986**	**0.978**	−0.769	**−0.999**	**−0.970**	**0.997**
Salvianolic acid B	**0.952**	**0.918**	**−0.982**	**−0.995**	**0.998**	**−0.973**	**−0.995**	**0.950**	**−0.921**
7α-hydroxyallyl-royleanone	−0.725	0.623	0.509	**−0.997**	**0.964**	**0.971**	**−0.959**	**0.994**	**−0.998**
Tanshinone IIB	0.140	−0.340	0.759	**−0.969**	**0.970**	**0.949**	**−0.997**	**0.998**	**0.968**
15,16′-dihydrotanshinone I	−0.423	0.768	**0.942**	**−0.973**	**0.973**	**0.989**	**−0.941**	**0.994**	**0.991**
Methyltanshinonate	0.018	**0.986**	**0.975**	**−0.980**	**0.984**	**0.989**	0.136	**0.995**	**0.999**
1,2′-dihydrotanshinone I	0.769	**−0.980**	0.611	**−0.968**	**0.983**	**0.969**	**0.991**	**0.995**	**0.994**
Tanshinone IIA	0.217	0.046	**0.909**	**−0.981**	**0.982**	**0.932**	0.894	**0.991**	**0.991**

[a] Positive and negative signs indicate positive and negative correlation in the concentration, respectively. The coefficient of 0.90 was used as the cutoff value based on the significant difference evaluation ($p < 0.001$).

Table S5. *P*-value of *S. miltiorrhiza* extracts from different genotypes for each locations (1, Genotype 1; 2, Genotype 2; 3, Genotype 3; 4, Genotype 4).

Putative Annotation	Zhuyang			Changiqng			Taian		
	1/2	2/3	3/4	1/2	2/3	3/4	1/2	2/3	3/4
Procatechuic acid	4.3×10^{-11} [a],*	1.8×10^{-8} *	1.6×10^{-14} *	—	—	—	2.1×10^{-10} *	1.4×10^{-4} *	4.5×10^{-16} *
Salvianolic acid F	2.9×10^{-13} *	1.7×10^{-8} *	3.7×10^{-7} *	4.1×10^{-12} *	4.1×10^{-12} *	6.7×10^{-15} *	1.0×10^{-20} *	2.5×10^{-8} *	9.9×10^{-10} *
Salvianolic acid I	4.4×10^{-26} *	1.1×10^{-10} *	3.5×10^{-11} *	—	4.1×10^{-18} *	1.2×10^{-1}	2.0×10^{-7} *	6.3×10^{-5} *	4.5×10^{-14} *
Salvianolic acid E	1.6×10^{-8} *	3.1×10^{-1}	4.0×10^{-6} *	3.8×10^{-7} *	2.9×10^{-11} *	8.0×10^{-16} *	3.0×10^{-17} *	1.1×10^{-11} *	1.3×10^{-8} *
Rosmarinic acid	1.7×10^{-11} *	9.5×10^{-7} *	2.3×10^{-19} *	1.0×10^{-12} *	2.1×10^{-7} *	2.3×10^{-7} *	3.0×10^{-17} *	1.1×10^{-3}	8.0×10^{-3}
Lithospermic acid	2.1×10^{-12} *	2.1×10^{-8} *	1.4×10^{-18} *	5.3×10^{-10} *	2.7×10^{-14} *	1.3×10^{-12} *	5.1×10^{-16} *	2.7×10^{-6} *	3.1×10^{-7} *
Prolithospermic acid derivative	8.4×10^{-5} *	5.4×10^{-4} *	8.3×10^{-2}	7.3×10^{-10} *	1.2×10^{-8} *	2.8×10^{-2}	3.1×10^{-21} *	3.1×10^{-4} *	2.3×10^{-13} *
Salvianolic acid B	1.5×10^{-4} *	4.8×10^{-7} *	1.8×10^{-12} *	4.1×10^{-11} *	3.2×10^{-14} *	1.1×10^{-10} *	5.7×10^{-15} *	1.4×10^{-4} *	2.5×10^{-4} *
7α-hydroxyallyl–royleanone	2.5×10^{-2}	1.4×10^{-2}	1.1×10^{-3}	1.3×10^{-12} *	5.2×10^{-6} *	5.7×10^{-10} *	3.6×10^{-9} *	2.1×10^{-4} *	7.7×10^{-5} *
Tanshinone IIB	1.5×10^{-2}	1.5×10^{-1}	4.1×10^{-2}	4.3×10^{-4} *	1.0×10^{-4} *	4.2×10^{-8} *	6.6×10^{-13} *	1.1×10^{-19} *	2.3×10^{-9} *
15,16′-dihydrotanshinone I	8.6×10^{-1}	2.6×10^{-1}	1.8×10^{-4} *	9.4×10^{-8} *	2.1×10^{-7} *	7.5×10^{-12} *	5.3×10^{-6} *	7.7×10^{-11} *	6.9×10^{-13} *
Methyltanshinonate	1.3×10^{-2}	2.0×10^{-4} *	5.9×10^{-4} *	3.1×10^{-4} *	4.7×10^{-11} *	6.8×10^{-15} *	2.3×10^{-1}	3.4×10^{-14} *	5.5×10^{-21} *
1,2′-dihydrotanshinone I	2.2×10^{-3}	4.1×10^{-4} *	2.1×10^{-1}	1.8×10^{-5} *	1.1×10^{-10} *	3.8×10^{-10} *	2.1×10^{-4} *	6.3×10^{-4} *	4.6×10^{-16} *
Tanshinone IIA	2.3×10^{-1}	1.6×10^{-1}	3.4×10^{-4} *	4.4×10^{-7} *	2.4×10^{-9} *	3.0×10^{-7} *	1.5×10^{-2}	2.3×10^{-4} *	2.2×10^{-15} *

[a] Multiple testing was corrected by Bonferroni correction. *$p < 7.57 \times 10^{-4}$ (0.05/66).

Table S6. Unidentified compounds in *S. miltiorrhiza* samples.

Peak No.	Unidentified Metabolites	Molecular Weight	λ_{max} (nm)	RT (min)	Negative Ion Mode		Positive Ion Mode		
					$[M-H]^-$	Major Fragments	$[M+Na]^+$	$[M+H]^+$	Major Fragments
3	U1	226	294–310	5.20			249	227	
8	U2	418	316–345–358–371	17.40	417			419	397
9	U3	339	319	17.60	338		362	340	
11	U4	452	251–288–317	19.20			475	453	
12	U5	162	212–282–324	19.60				163	
19	U6	396	212–255–280	26.60			419	397	
22	U7	718	285–310	28.49	717			719	
25	U8	301	285–310–330	32.51			324	302	346
27	U9	296	260–310	35.62	295				
28	U10	298	285–320	36.35	297			299	
31	U11	298	285–315–380	42.24	297				
35	U12	340	255–270–380	45.37			363	341	
38	U13	318	250–380	45.92			341	319	
41	U14	488	250(sh)–380	46.69	487				
43	U15	308	255–350–380	47.35			331	309	
45	U16	358	245(sh)	48.12			381	359	
51	U17	314	250–280–360	50.31	313				
54	U18	314	250–350–360	53.82	313				
56	U19	280	250	56.65				281	
57	U20	331	255–270–360–380	56.97			354	332	
58	U21	279	250–320–330–360	57.72			310	280	
61	U22	273	255–335–360	59.44			296	274	256
63	U23	317	255–280–410	60.76				318	340
64	U24	314	275–410–440–255	61.26	313	269, 213			
65	U25	358	255–280	62.70			381	359	
66	U26	477	255	63.10			500	478	

Table S7. Percentage of paired locations (12) and paired genotypes (18) with relative abundances that were statistically significance ($p < 7.57 \times 10^{-4}$).

Metabolites	Percentage of Paired Locations [a]	Percentage of Paired Genotypes[b]	Metabolites	Percentage of Paired Locations	Percentage of Paired Genotypes	Metabolites	Percentage of Paired Locations	Percentage of Paired Genotypes
Danshensu	67%	56%	Tormentic acid	58%	44%	Dehydromiltirone	8%	6%
Procatechuic acid	67%	**61%**	Trijuganone C	**75%**	33%	Procatechu aldehyde	25%	33%
Salvianolic acid F	**67%** [c]	**100%**	Danshenxinkun A	75%	33%	Caffeic acid	33%	33%
Salvianolic acid I	**92%**	**78%**	Przewa tanshinone A	50%	39%	Isosalvianolic acid B	33%	33%
Salvianolic acid E	**100%**	**83%**	Trijuganone B	**75%**	39%	Salvianolic acid L	42%	33%
Rosmarinic acid	**75%**	**72%**	Cryptotanshinone	**92%**	33%	Salvianolic acid A	33%	39%
Luteolin	75%	50%	Tanshinone I	**83%**	44%	Tanshindiol C	42%	44%
Lithospermic acid	**100%**	**100%**	Vanillic acid	42%	50%	Royleanone-4	42%	44%
Prolithospermic acid derivative	**75%**	**72%**	Ferulic acid	25%	50%	1-Ketoisocryptotanshinone	42%	28%
Salvianolic acid B	**100%**	**100%**	7α-Hydroxyallyl−royleanone	25%	**78%**	Neocryptotanshinone	33%	22%
Tanshinone IIB	**92%**	**61%**	Trijuganone A	17%	39%	Miltipolone	42%	39%
β-Sitosterol	67%	50%	Miltirone	17%	33%			
Tanshinonealdehyde	75%	50%						
15,16-Dihydrotanshinone I	**100%**	**78%**						
Methyltanshinonate	**100%**	**78%**						
1,2-Dihydrotanshinone I	**75%**	**72%**						
Tanshinone IIA	**92%**	**56%**						

[a] Multiple testing was corrected by Bonferroni correction. Bonferroni−corrected p value for significance: 7.57×10^{-4} (0.05/66).[a] Paired locations (12) were ZY *vs.* CQ, ZY *vs.* TA, CQ *vs.* TA in four genotypes. [b] Paired genotypes (18) were Genotype 1 *vs.* Genotype 2, Genotype 1 *vs.* Genotype 3, Genotype 1 *vs.* Genotype 4, Genotype 2 *vs.* Genotype 3, Genotype 2 *vs.* Genotype 4, Genotype 3 *vs.* Genotype 4 at three locations. [c] The locations−specific and genotypes−specific metabolites were labeled by bold fonts.

(a) Root yield

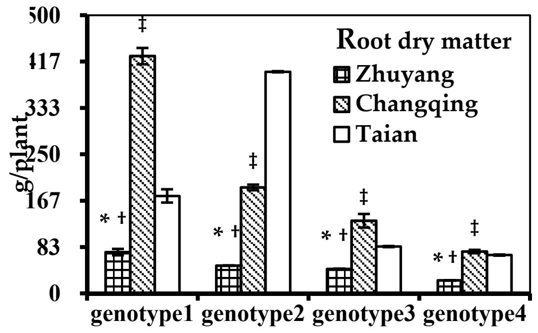

(b) Activities of four enzymes

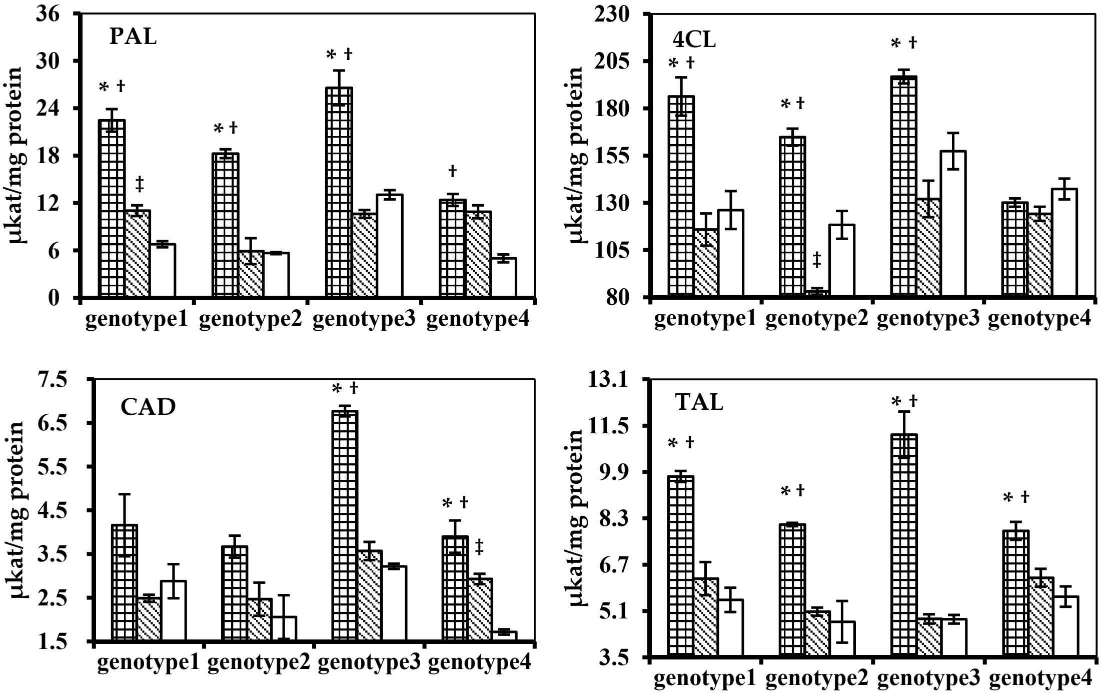

Figure S1. Root yield (**a**), and activities of four enzymes (**b**) in *S. miltiorrhiza* in different locations. F our enzymes include PAL, 4CL, CAD, TAL. * Significant difference according to Bonferroni correction ($p < 0.00033$) Zhuyang *vs.* Changiqng; † Significant difference according to Bonferroni correction ($p < 0.00033$) Zhuyang *vs.* Taian; ‡ Significant difference according to Bonferroni correction ($p < 0.00033$) Changqing *vs.* Taian; PAL, phenylalanine ammonia-lyase; 4CL, 4-coumarate-CoA ligase; CAD, cinnamyl alcohol–NADPH dehydrogenase; TAL, tyrosine ammonic–lyase.

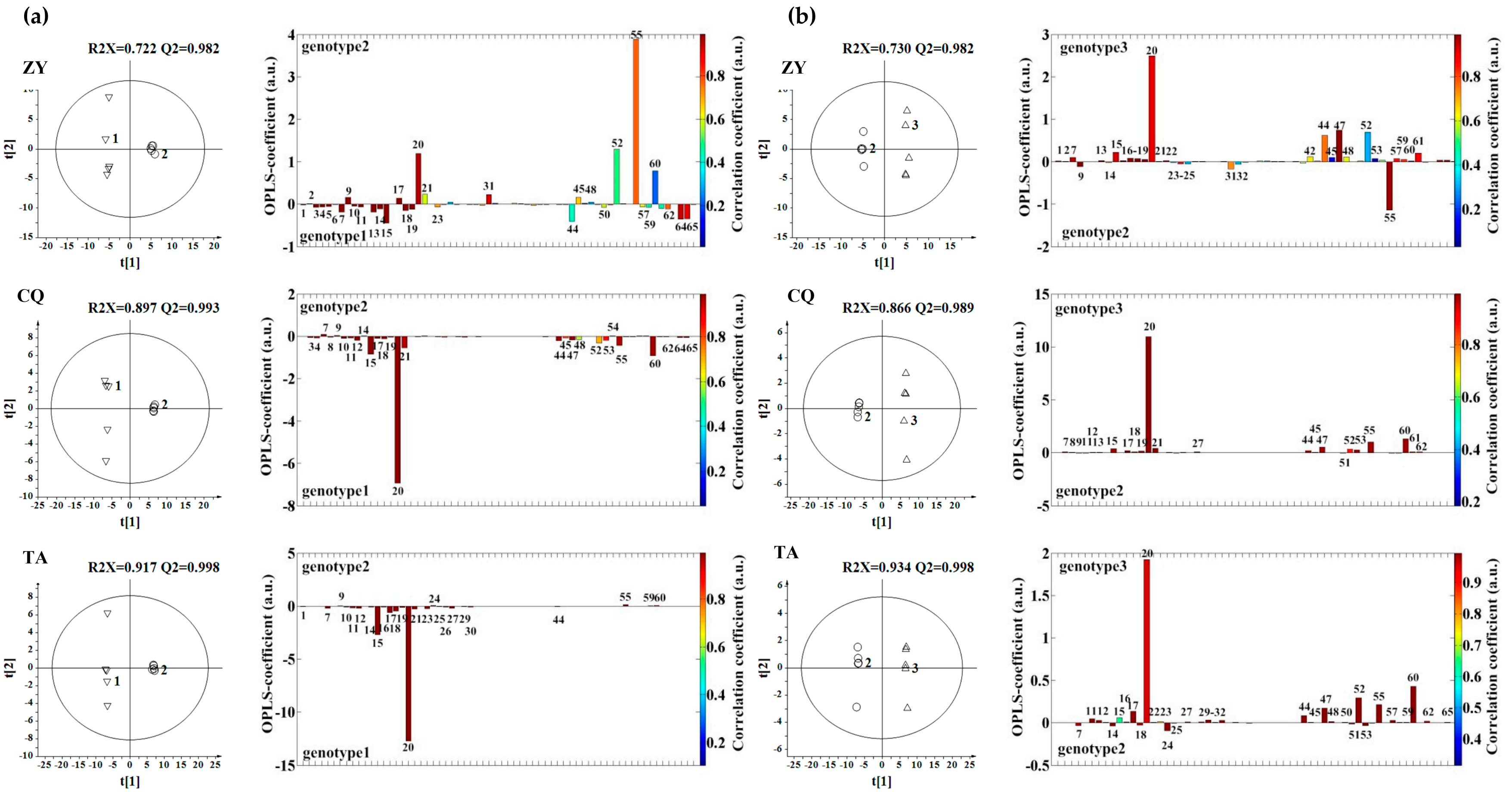

Figure S2. *Cont.*

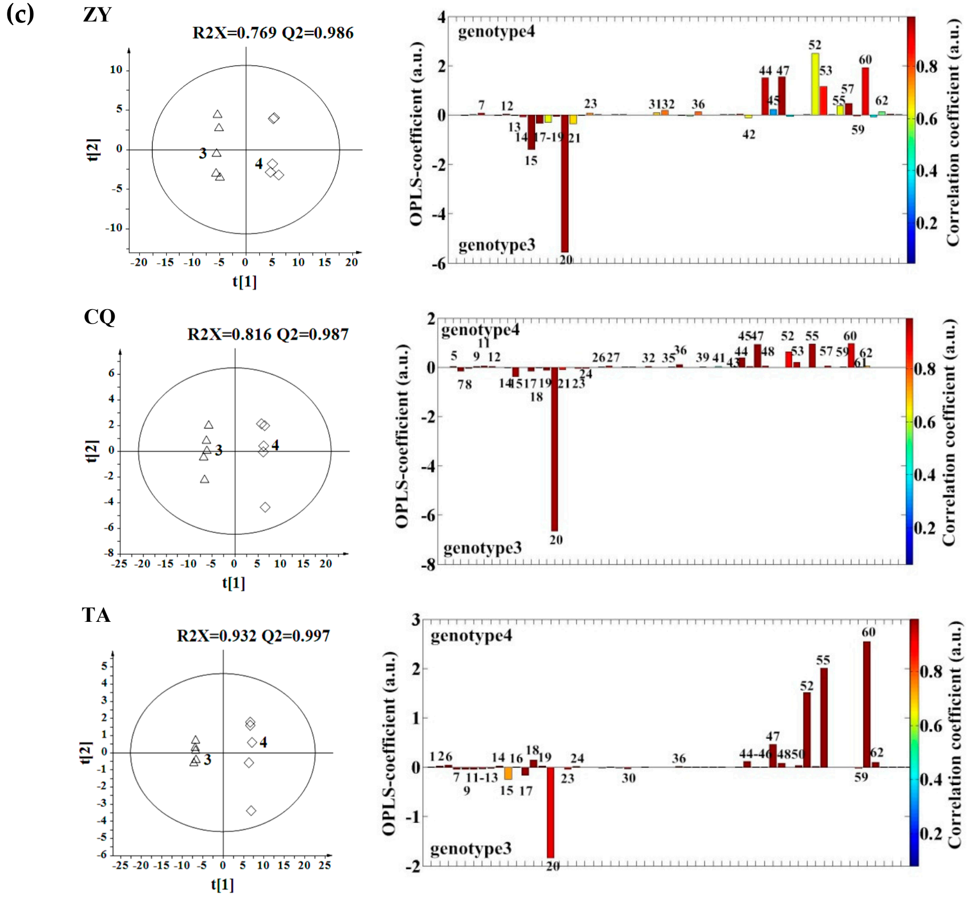

Figure S2. OPLS-DA scores plots (left) and coefficient−coded loadings plots (right) deriver from LC-MS data for *S. miltiorrhiza* extracts obtained from four different genotypes (1, Genotype 1; 2, Genotype 2; 3, Genotype 3; 4, Genotype 4)for each location (ZY, Zhuyang; CQ, Changqing; TA, Taian): 2, Procatechuic acid; 7, Salvianolic acid F; 13, Salvianolic acid I; 14, Salvianolic acid E; 15, Rosmarinic acid; 17, Lithospermic acid; 18, Prolithospermic acid derivative; 20, Salvianolic acid B; 30, 7α−hydroxyallyl−royleanone; 32, Tanshinone ⅡB; 44, 15,16′−dihydrotanshinone Ⅰ; 47, Methyltanshinonate; 55, 1,2′-dihydrotanshinone Ⅰ; 60, Tanshinone ⅡA. (**a**), *S. miltiorrhiza* extracts obtained from Genotype 1 *vs.* Genotype 2 for each location; (**b**), *S. miltiorrhiza* extracts obtained from Genotype 2 *vs.* Genotype 3 for each location; (**c**), *S. miltiorrhiza* extracts obtained from Genotype 3 *vs.* Genotype 4 for each location.

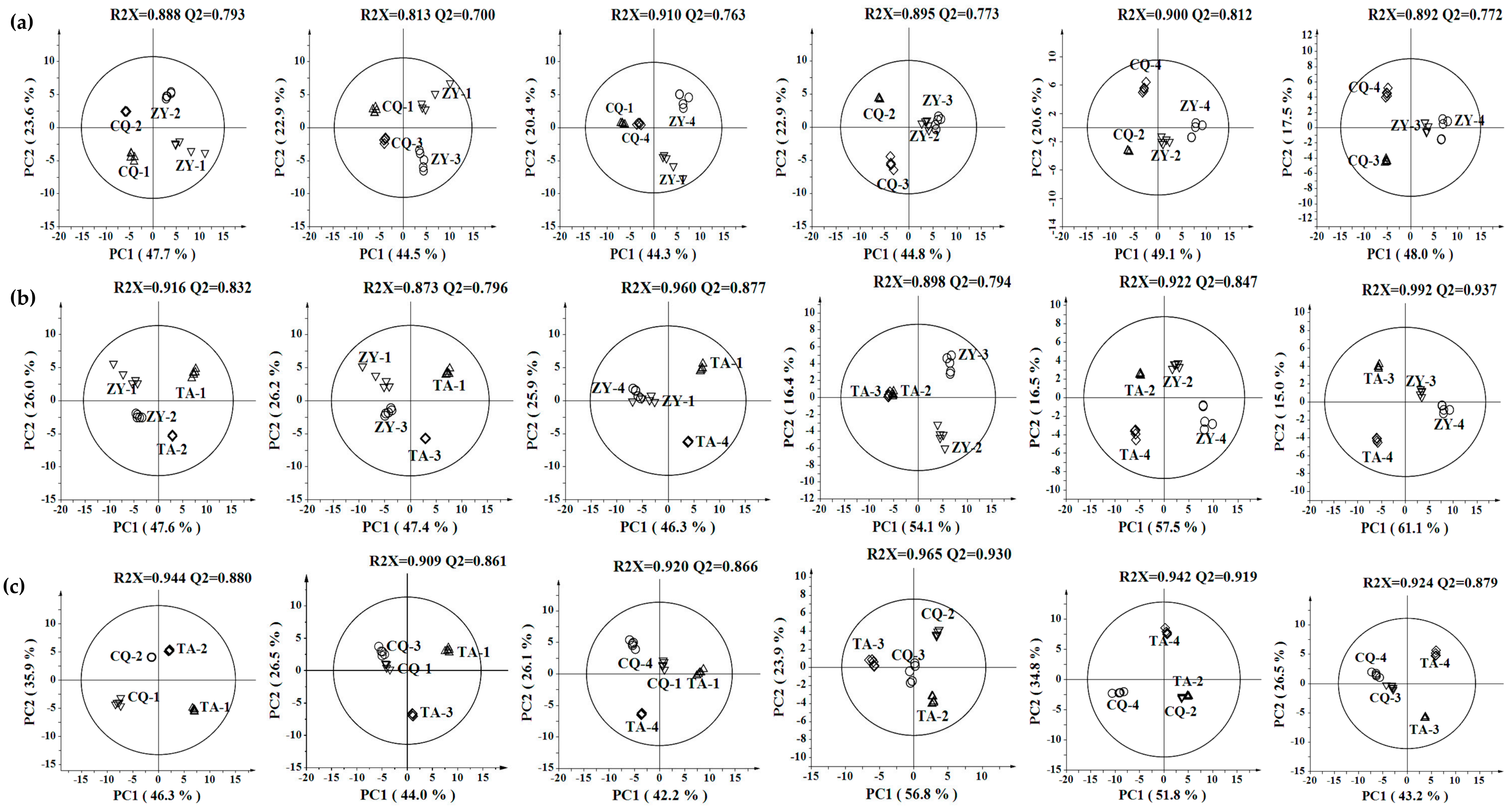

Figure S3. PCA scores plots derived from LC-MS data for *S. miltiorrhiza* extracts obtained from paired locations and genotypes. (ZY, Zhuyang; CQ, Changqing; TA, Taian. 1, Genotype 1; 2, Genotype 2; 3, Genotype 3; 4, Genotype 4). (a), *S. miltiorrhiza* extracts obtained from ZY *vs.* CQ; (b), *S. miltiorrhiza* extracts obtained from ZY *vs.* TA; (c), *S. miltiorrhiza* extracts obtained from CQ *vs.* TA.

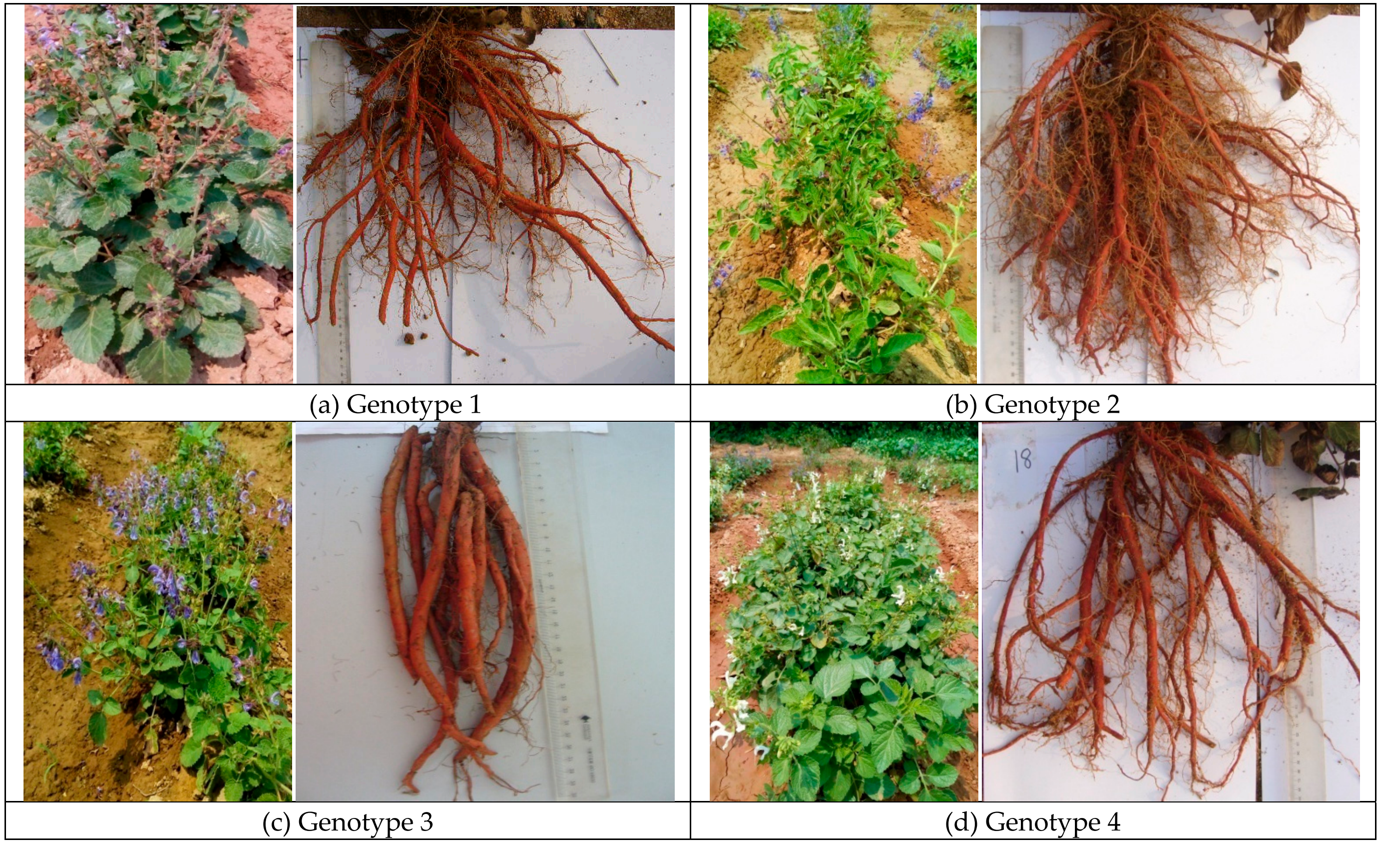

Figure S4. Plants and roots of four genotypes (**a**, Genotype 1; **b**, Genotype 2; **c**, Genotype 3; **d**, Genotype 4).

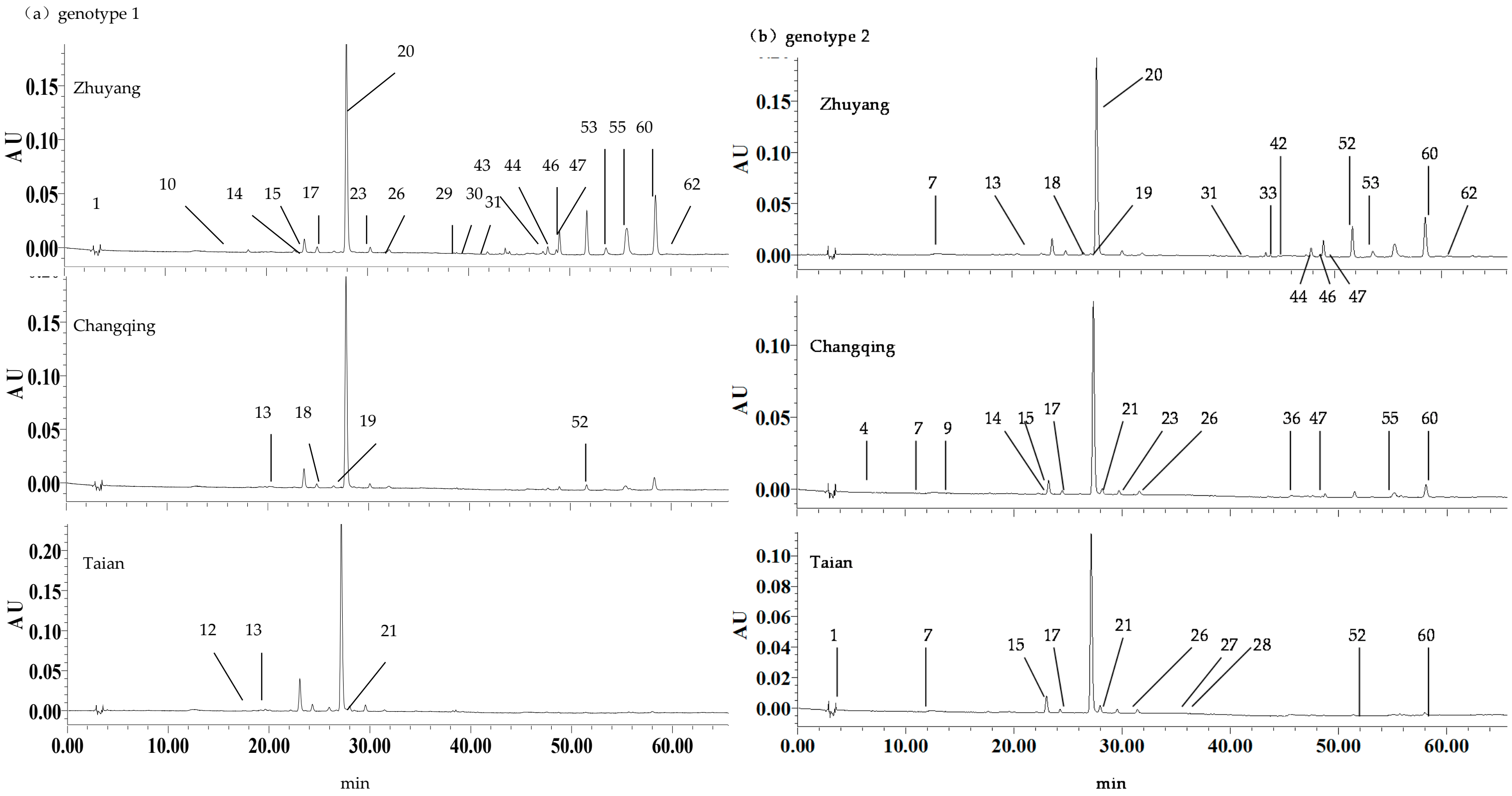

Figure S5. *Cont.*

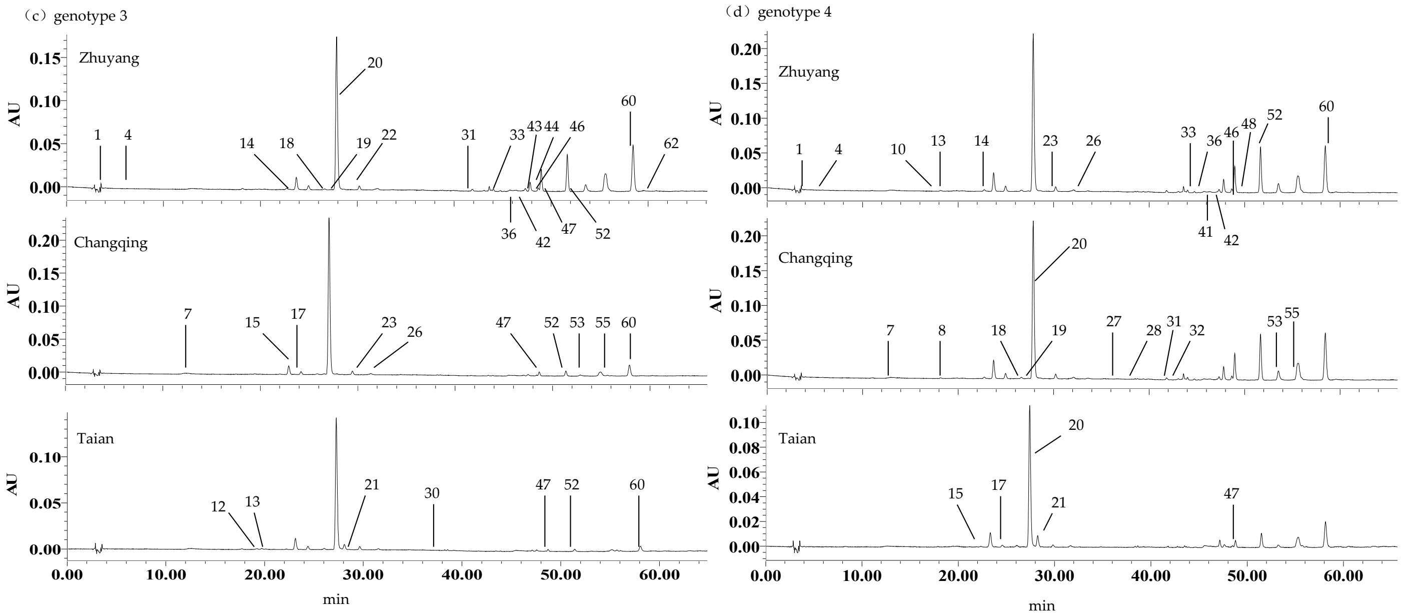

Figure S5. LC−UV chromatograms (280nm) for *S. miltiorrhiza* from three different locations (Zhuyang, Changqing, Taian) for each genotype: 1, Danshensu; 4, Procatechuic aldehyde; 7, Salvianolic acid F; 8, U2; 9, U3; 10, Ferulic acid; 12, U5; 13, Salvianolic acid I; 14, Salvianolic acid E; 15, Rosmarinic acid; 17, Lithospermic acid; 18, Prolithospermic acid derivative; 19, U6; 20, Salvianolic acid B; 21, Isosalvianolic acid B; 22, U7; 23, Salvianolic acid L; 24, Salvianolic acid A; 26, Tanshindiol C; 27, U9; 28, U10; 29, Royleanone−4; 30, 7α−hydroxyallyl−royleanone; 31, U11; 32, Tanshinone ⅡB; 33, 1-ketoisocryptotanshinone; 36, Trijuganone C; 41, U14; 42, Przewa tanshinone A; 43, U15; 44, 15,16′-dihydrotanshinone Ⅰ; 46, Neocryptotanshinone; 47, Methyltanshinonate; 48, Trijuganone B; 52, Cryptotanshinone; 53, Tanshinone Ⅰ; 55, 1,2′-dihydrotanshinone Ⅰ; 60, Tanshinone ⅡA; 62, Miltirone. (**a**), LC−UV chromatograms from three different locations for Genotype 1; (**b**), LC-UV chromatograms from three different locations for Genotype 2; (**c**), LC-UV chromatograms from three different locations for Genotype 3; (**d**), LC-UV chromatograms from three different locations for Genotype 4.

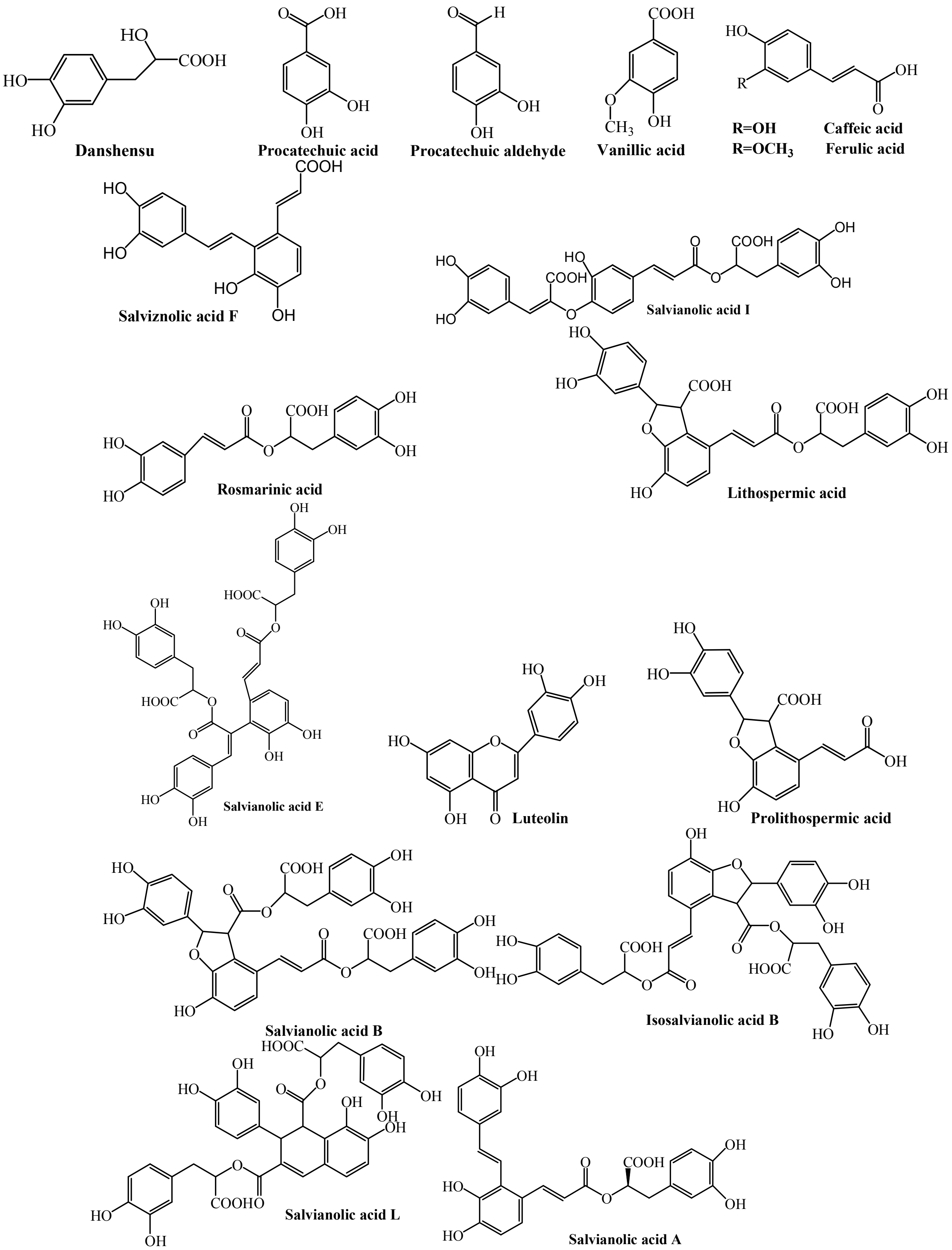

Figure S6. *Cont.*

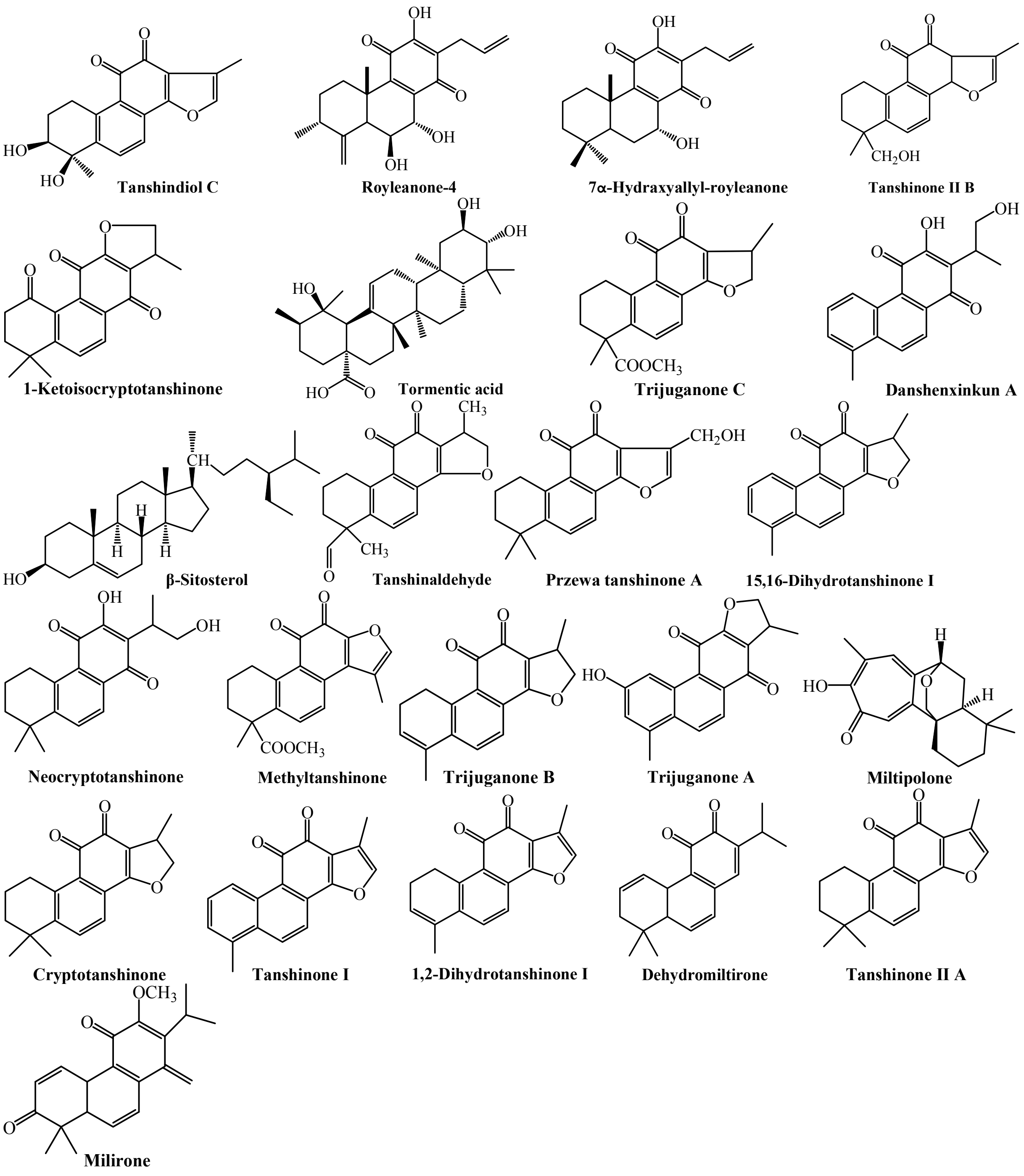

Figure S6. The structures of some secondary metabolites in *S. miltiorrhiza*.

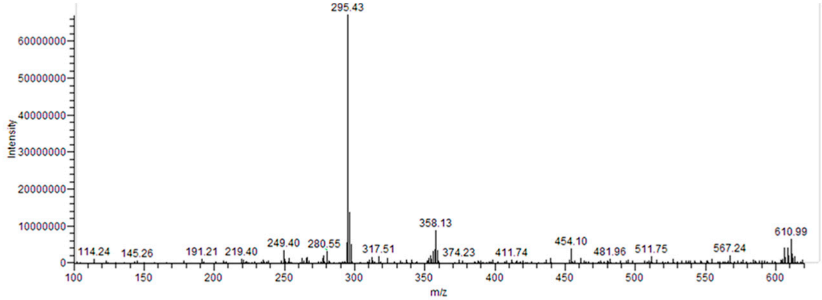

(1) Tanshinone ⅡA positive ion mode

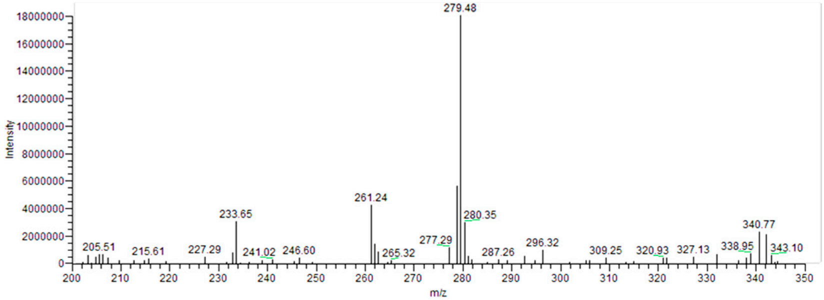

(2) 1,2′−dihydrotanshinone Ⅰ positive ion mode

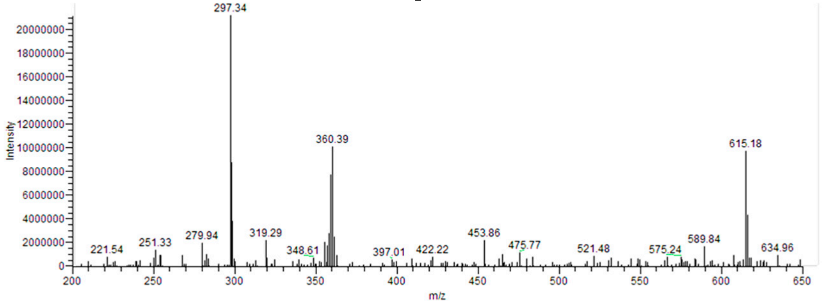

(3) Cryptotanshinone positive ion mode

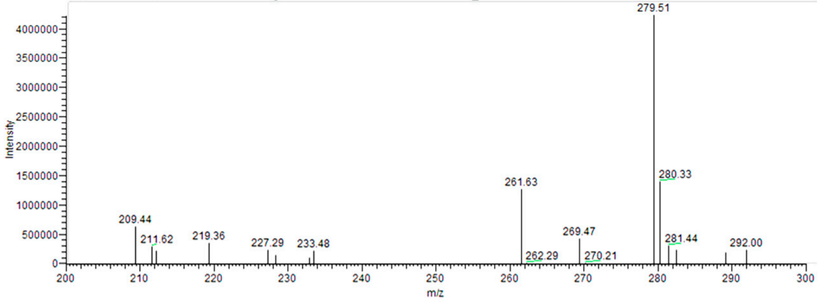

(4) 15,16′−dihydrotanshinone Ⅰ positive ion mode

Figure S7. *Cont.*

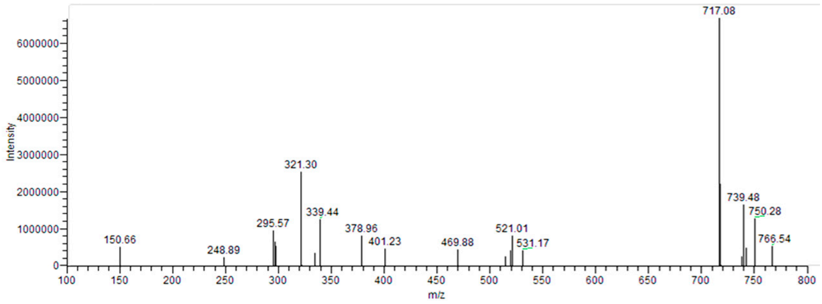

(5) Salvianolic acid B negative ion mode

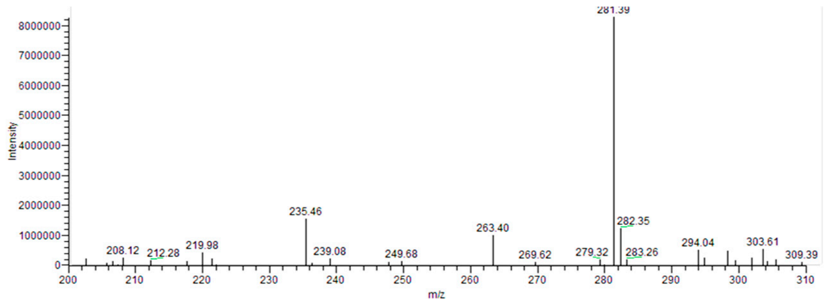

(6) Trijuganone B positive ion mode

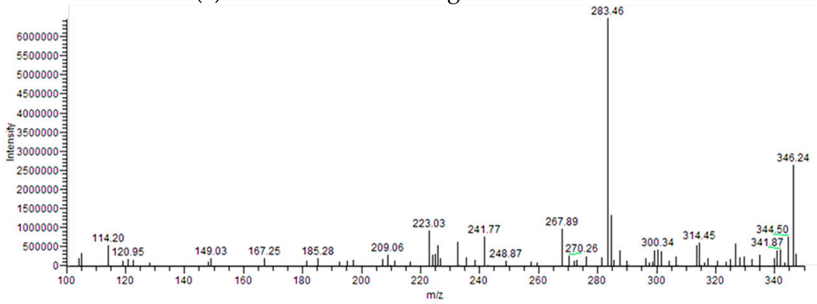

(7) Miltirone positive ion mode

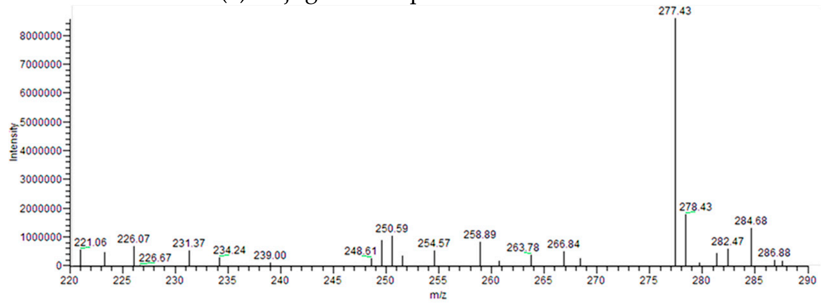

(8) Tanshinone Ⅰ positive ion mode

Figure S7. Some spectra of *S. miltiorrhiza* compounds in our studies: (**1**), tanshinone IIA; (**2**), 1,2′−dihydrotanshinone I; (**3**), cryptotanshinone; (**4**), 15,16′−dihydrotanshinone I; (**5**), salvianolic acid B; (**6**), trijuganone B; (**7**), miltirone; (**8**), tanshinone I.
